# Supplementary figures and images for: Investigating variability in microbial community composition in replicate environmental DNA samples down lake sediment cores
Source: PLoS One. 2021 May 3;16(5):e0250783. doi: 10.1371/journal.pone.0250783 (PMC8092796; doi:10.1371/journal.pone.0250783)

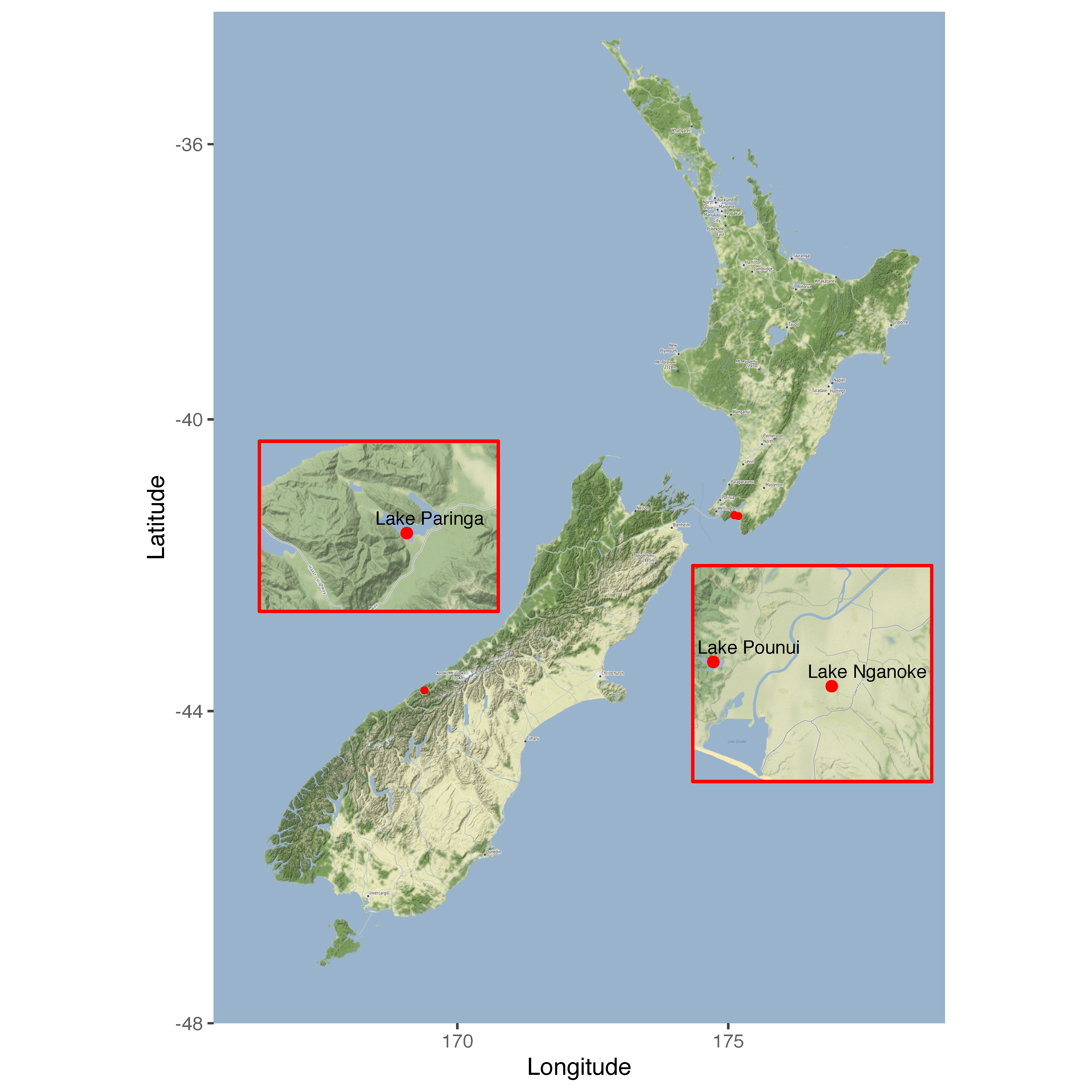

Supplement: S1 Fig — The map was produced in ggmap [32] using Map tiles by Stamen Design, under CC BY 3.0. Data by OpenStreetMap, under ODbL. (TIF) [file pone.0250783.s001.tif]

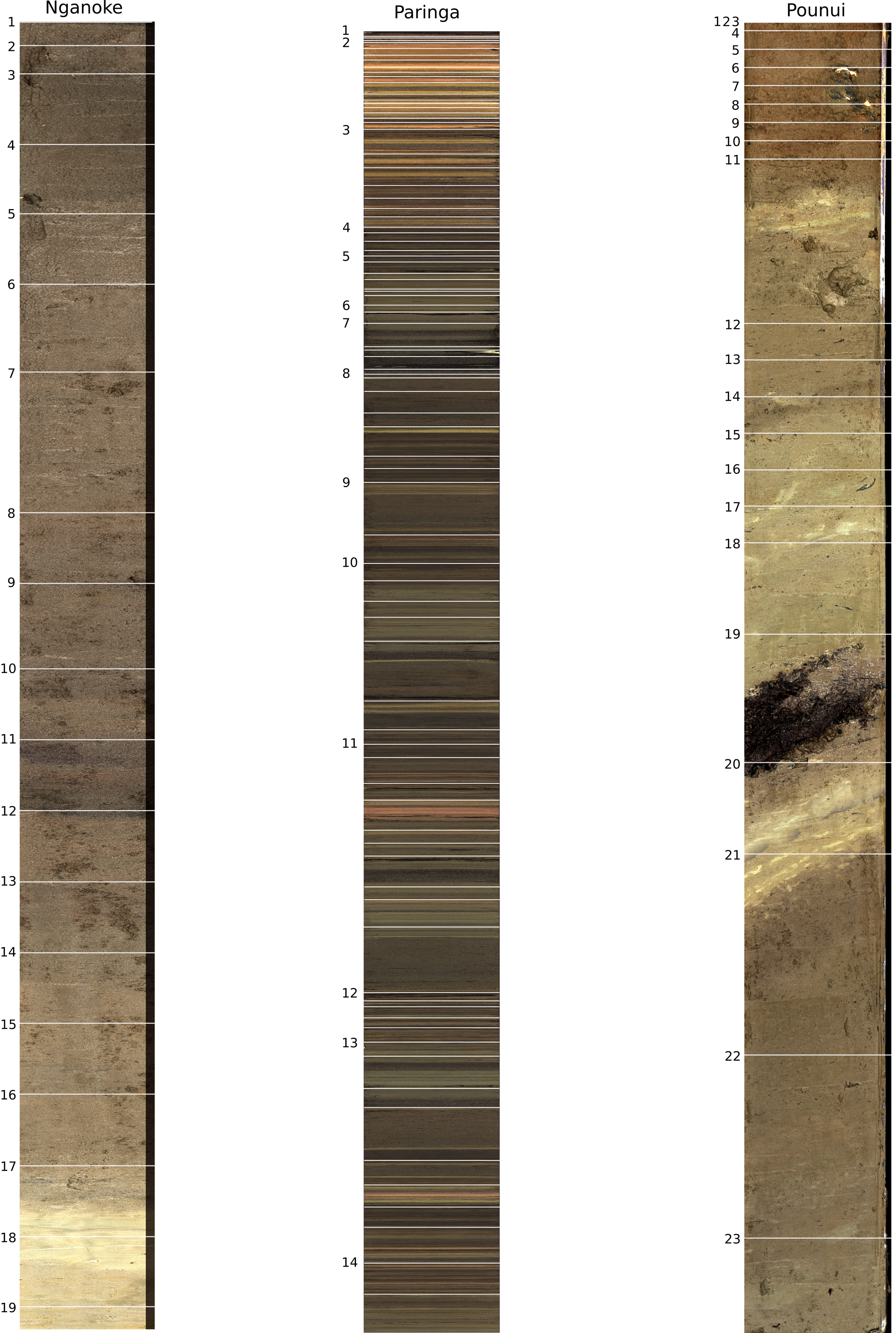

Supplement: S2 Fig — (TIF) [file pone.0250783.s002.tif]

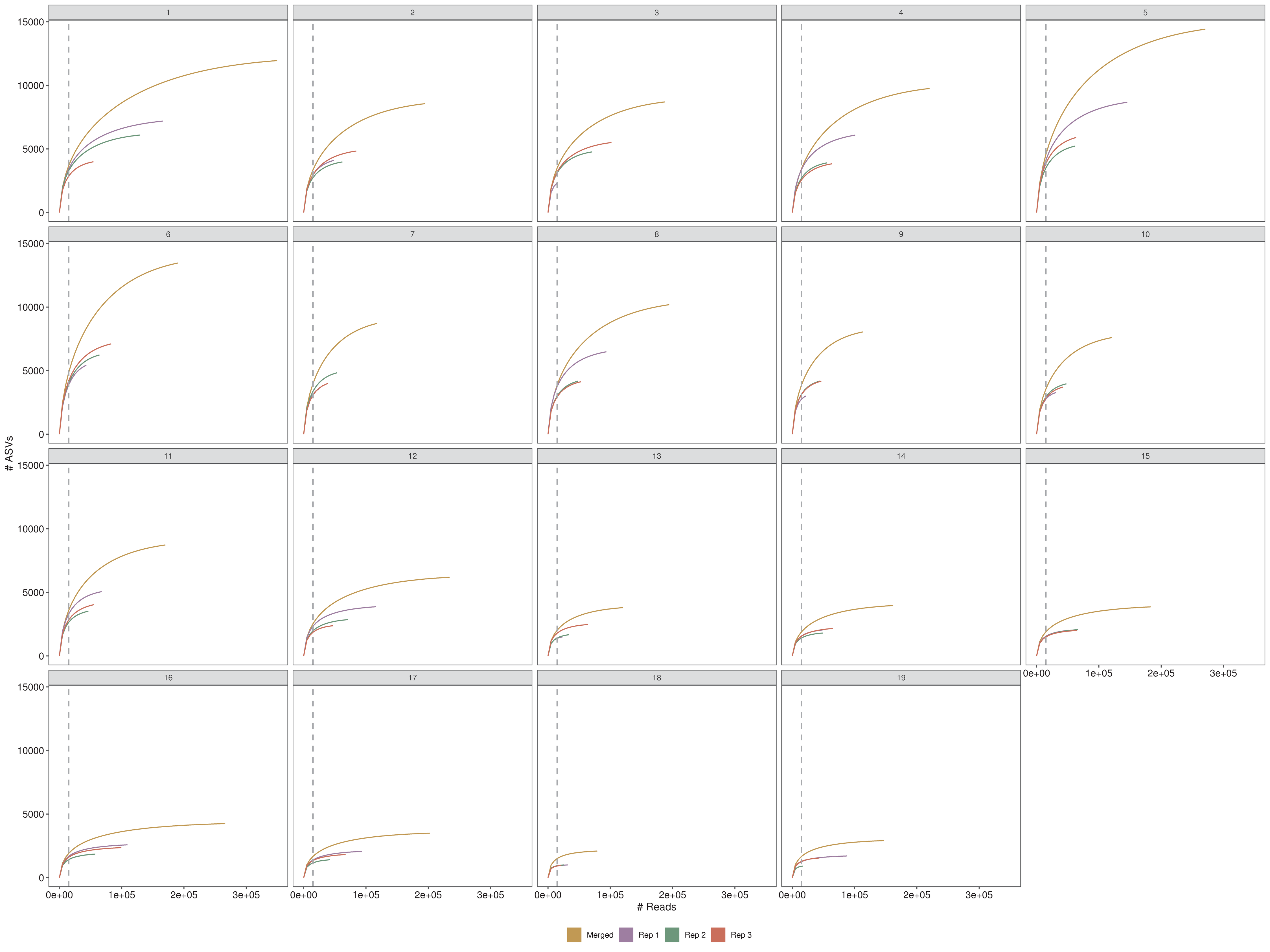

Supplement: S3 Fig — (TIF) [file pone.0250783.s003.tif]

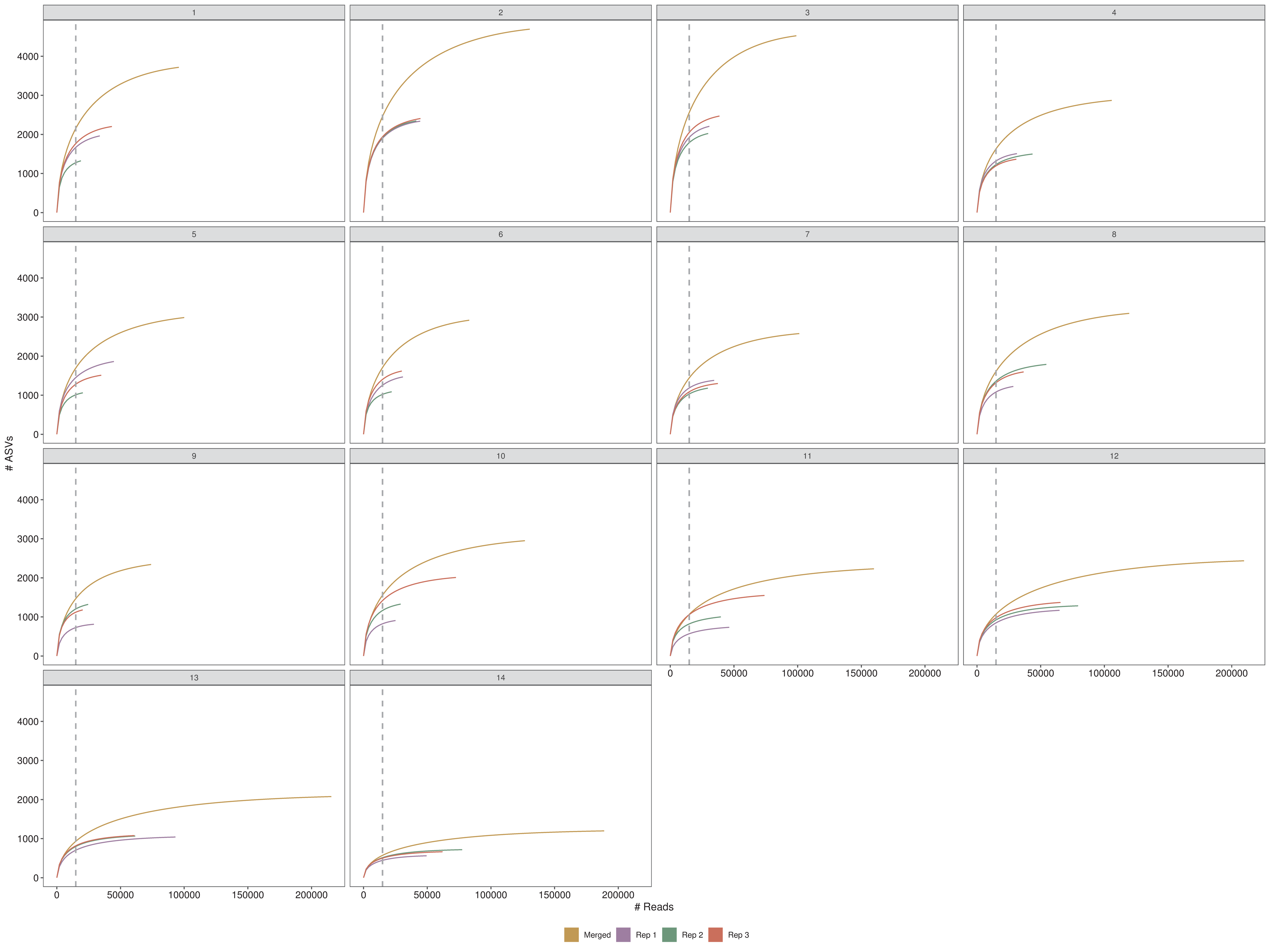

Supplement: S4 Fig — (TIF) [file pone.0250783.s004.tif]

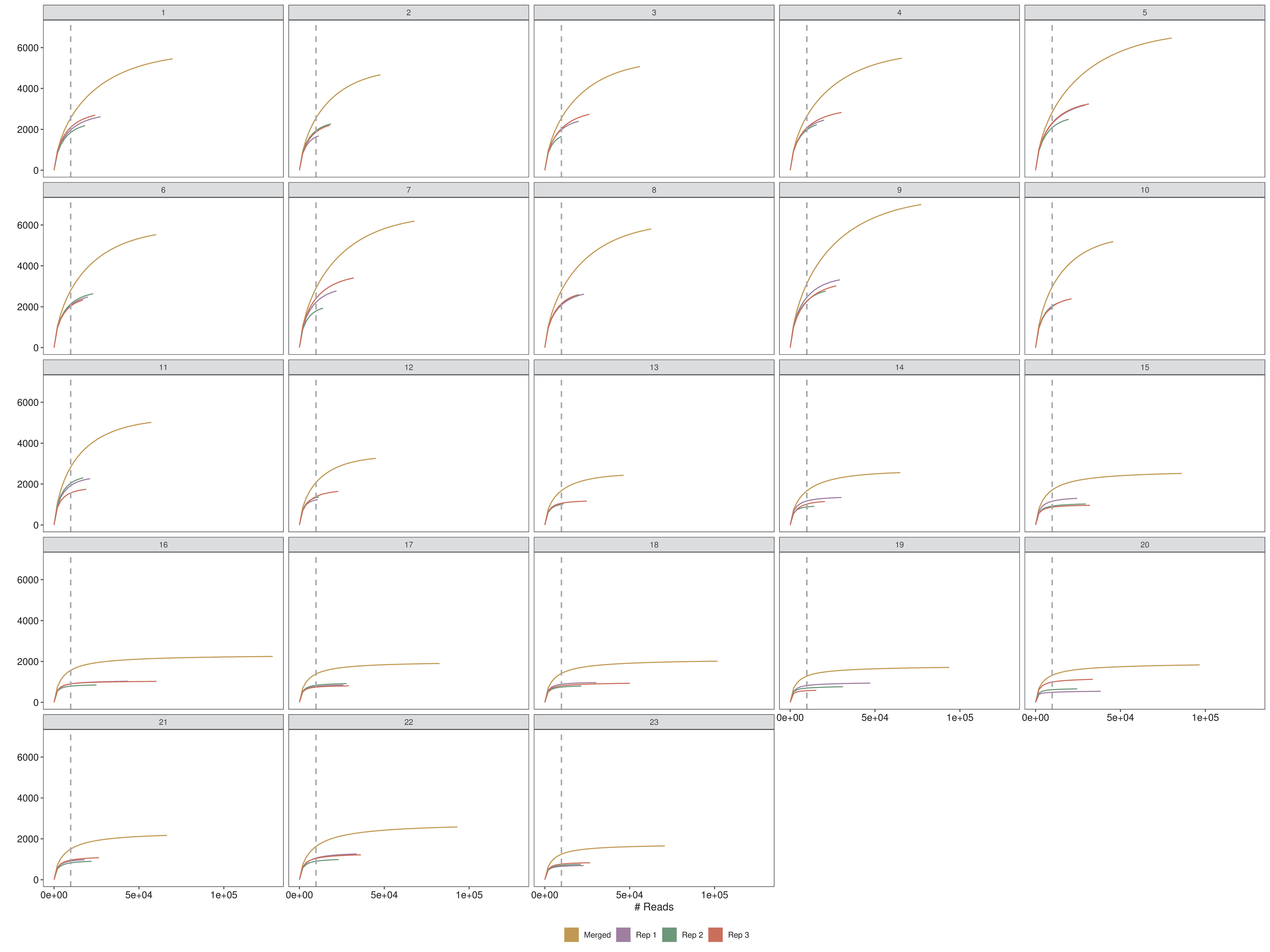

Supplement: S5 Fig — (TIF) [file pone.0250783.s005.tif]

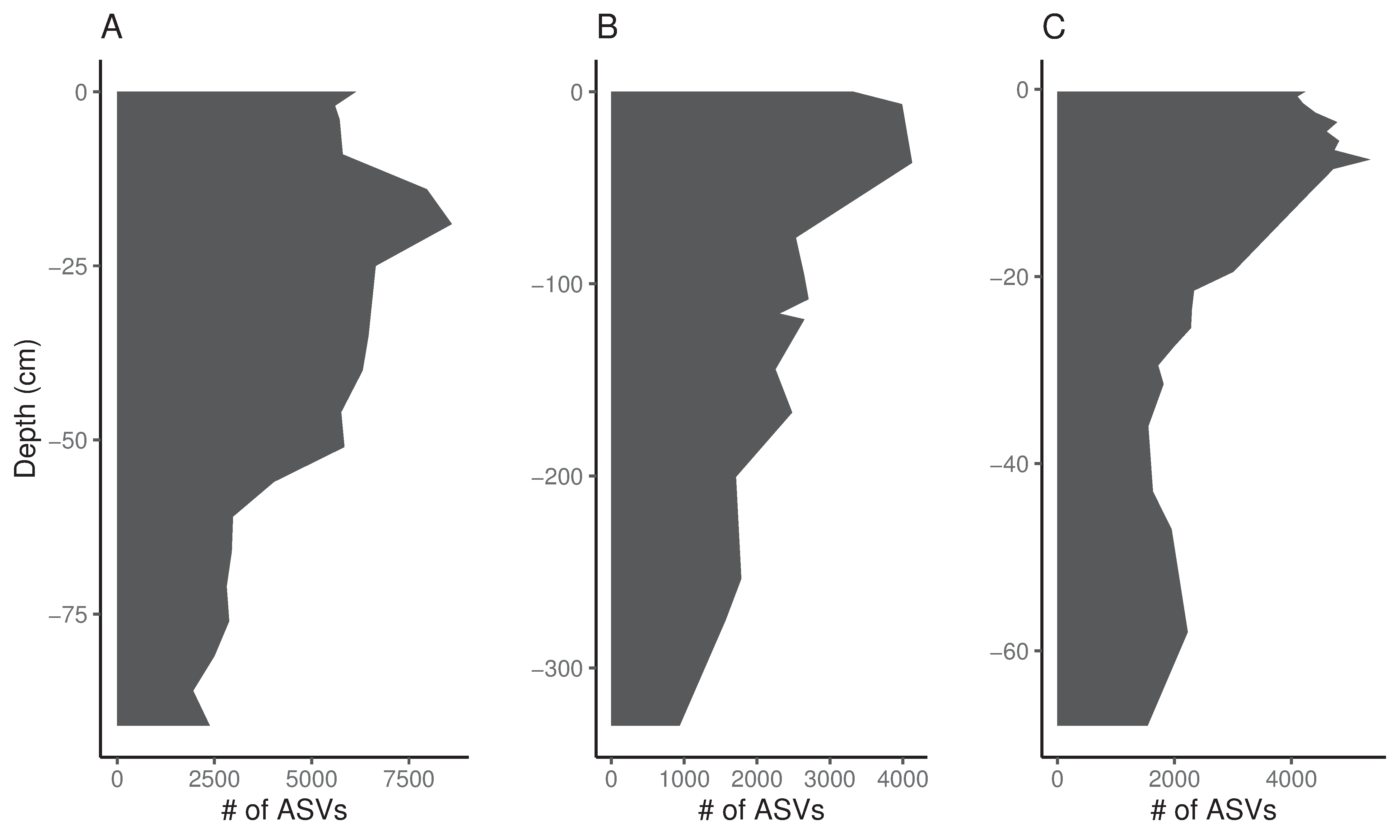

Supplement: S6 Fig — A—Lake Nganoke; B—Lake Paringa; C- Lake Pounui. (TIF) [file pone.0250783.s006.tif]

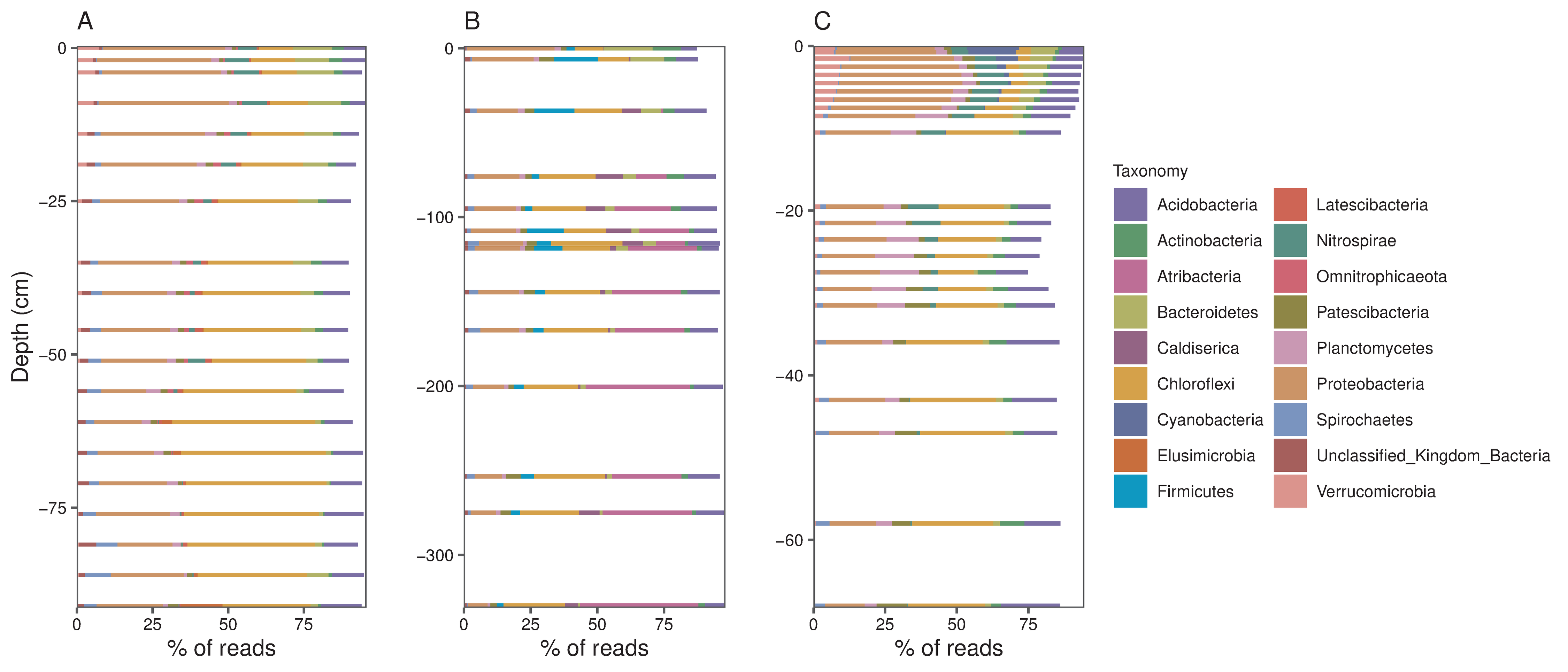

Supplement: S7 Fig — A—Lake Nganoke; B—Lake Paringa; C- Lake Pounui. Phyla that did not account for on average > 1% of the community were not show so bars do not add up to 100%. (TIF) [file pone.0250783.s007.tif]

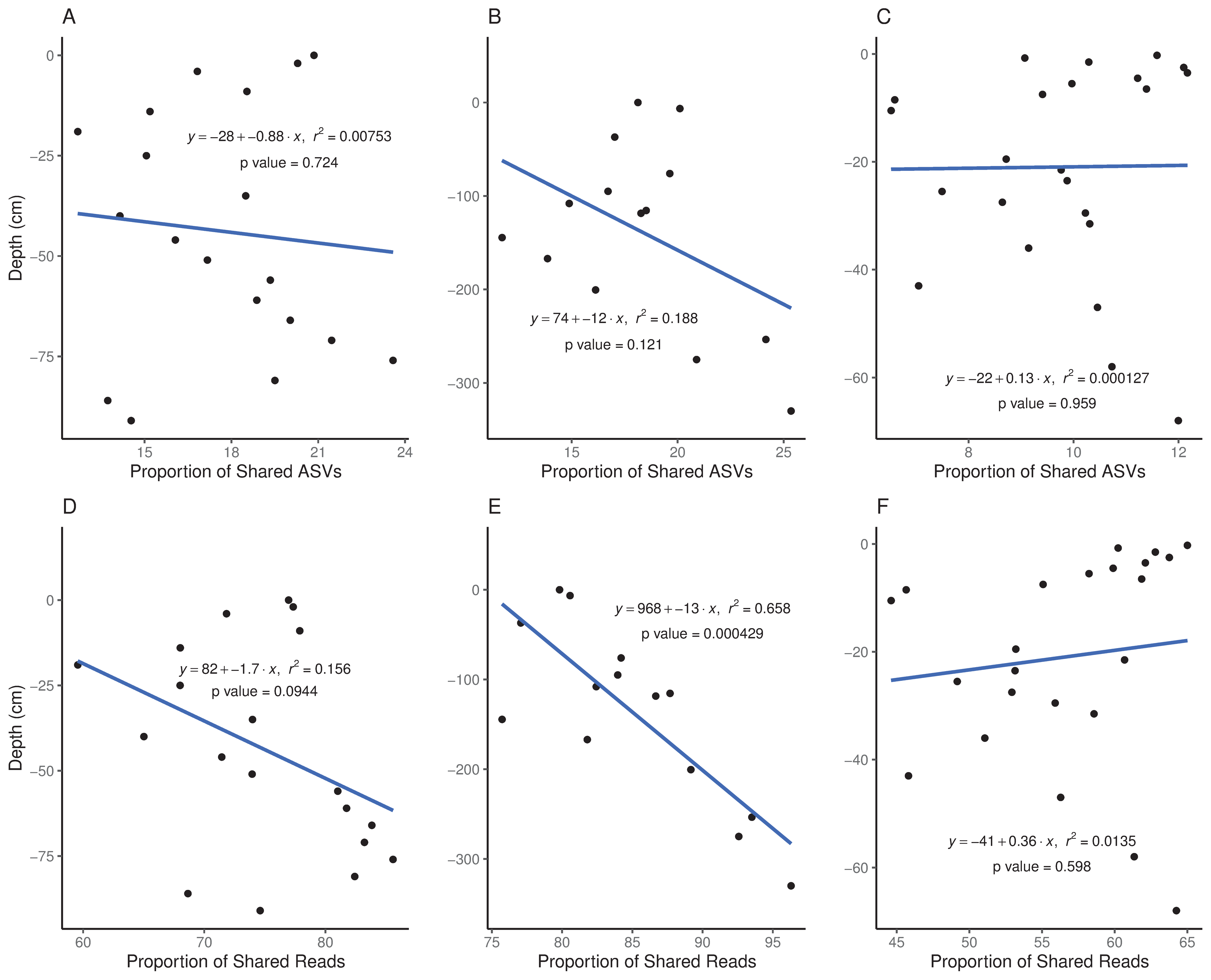

Supplement: S8 Fig — Note the different scales. (TIF) [file pone.0250783.s008.tif]
