# Supplementary material for: Investigating variability in microbial community composition in replicate environmental DNA samples down lake sediment cores
Source: PLoS One. 2021 May 3;16(5):e0250783. doi: 10.1371/journal.pone.0250783 (PMC8092796; doi:10.1371/journal.pone.0250783)
Supplement: Attachment — Submitted filename: PONE-D-20-32628_reviewer-2 +comments.pdf [file pone.0250783.s012.pdf]

PLOS ONE

Investigating variability in microbial community composition in replicate environmental DNA samples down lake sediment cores

--Manuscript Draft--

**Manuscript Number:**	PONE-D-20-32628
**Article Type:**	Research Article
**Full Title:**	Investigating variability in microbial community composition in replicate environmental DNA samples down lake sediment cores
**Short Title:**	Replicate variability in eDNA lake sediment core samples
**Corresponding Author:**	John K. Pearman, Ph.D<br>Cawthron Institute<br>Nelson, NEW ZEALAND
**Keywords:**	environmental DNA; bioinformatics; sediment core; bacteria
**Abstract:**	Lake sediments are natural archives that accumulate information about biological communities and their surrounding catchments. Paleolimnology has traditionally focussed on identifying fossilized organisms to reconstruct past environments. In the last decade, the application of molecular methodologies has increased in paleolimnological studies, but further studies investigating factors such as sample heterogeneity and DNA degradation are required. In the present study we investigated bacterial community heterogeneity (16S rRNA metabarcoding) within depth slices. Sediment cores were collected from three lakes with differing sediment compositions. Samples were collected from a variety of depths (1-cm width) which represent a period of time of approximately 1,200 years. Triplicate samples were collected from each slice and bacterial 16S rRNA metabarcoding was undertaken on each sample. Rarefaction curves showed that except for the deepest (oldest) slices, the combination of three replicate samples were insufficient to characterise the entire bacterial diversity. However, shared Amplicon Sequence Variants (ASVs) accounted for the majority of the reads in each slice (max. shared proportional read abundance 96%, 86%, 65% in the three lakes). Non-metric multidimensional scaling analysis showed that replicates within a slice generally clustered together. A comparison of the variability among replicates showed that there was high community dissimilarity in older sediment in one of the cores, which was likely due to the laminae in the sediment core not been horizontal. Given that most paleolimnology studies explore broad scale shifts in community structure, rather than seeking to identify rare species, this study demonstrates that a single sample is adequate to characterise shifts in dominant bacterial ASVs.
**Order of Authors:**	John K. Pearman, Ph.D
	Georgia Thomson-Laing
	Jamie D Howarth
	Marcus J Vandergoes
	Lucy Thompson
	Andrew Rees
	Susanna A Wood
**Additional Information:**	
**Question**	**Response**
**Financial Disclosure**<br><br>Enter a financial disclosure statement that describes the sources of funding for the work included in this submission. Review	This research was funded by the New Zealand Ministry of Business, Innovation and Employment research programme - Our lakes' health; past, present and future (C05X1707). The funders had no role in study design, data collection and analysis, decision to publish, or preparation of the manuscript.

the submission guidelines for detailed requirements. View published research articles from *PLOS ONE* for specific examples.<br><br>This statement is required for submission and **will appear in the published article** if the submission is accepted. Please make sure it is accurate.<br><br>**Unfunded studies**<br>Enter: *The author(s) received no specific funding for this work.*<br><br>**Funded studies**<br>Enter a statement with the following details:<br>• Initials of the authors who received each award<br>• Grant numbers awarded to each author<br>• The full name of each funder<br>• URL of each funder website<br>• Did the sponsors or funders play any role in the study design, data collection and analysis, decision to publish, or preparation of the manuscript?<br>• **NO** - Include this sentence at the end of your statement: *The funders had no role in study design, data collection and analysis, decision to publish, or preparation of the manuscript.*<br>• **YES** - Specify the role(s) played.<br><br>* typeset	
**Competing Interests**<br><br>Use the instructions below to enter a competing interest statement for this submission. On behalf of all authors, disclose any competing interests that could be perceived to bias this work—acknowledging all financial support and any other relevant financial or non-financial competing interests.<br><br>This statement **will appear in the published article** if the submission is accepted. Please make sure it is accurate. View published research articles from *PLOS ONE* for specific examples.	The authors have declared that no competing interests exist.

**NO authors have competing interests**<br><br>Enter: *The authors have declared that no competing interests exist.*<br><br>**Authors with competing interests**<br><br>Enter competing interest details beginning with this statement:<br><br>*I have read the journal's policy and the authors of this manuscript have the following competing interests: [insert competing interests here]*<br><br>* typeset	
**Ethics Statement**<br><br>Enter an ethics statement for this submission. This statement is required if the study involved:<br><br>• Human participants<br>• Human specimens or tissue<br>• Vertebrate animals or cephalopods<br>• Vertebrate embryos or tissues<br>• Field research<br><br>Write "N/A" if the submission does not require an ethics statement.<br><br>General guidance is provided below. Consult the submission guidelines for detailed instructions. **Make sure that all information entered here is included in the Methods section of the manuscript.**	The landowners at lakes Nganoke and Pounui are thanked for access to the launching sites. The Department of Conservation is acknowledged for assistance with permitting (Lake Paringa).

Powered by Editorial Manager® and ProduXion Manager® from Aries Systems Corporation

**Format for specific study types**<br><br>**Human Subject Research (involving human participants and/or tissue)**<br>• Give the name of the institutional review board or ethics committee that approved the study<br>• Include the approval number and/or a statement indicating approval of this research<br>• Indicate the form of consent obtained (written/oral) or the reason that consent was not obtained (e.g. the data were analyzed anonymously)<br><br>**Animal Research (involving vertebrate animals, embryos or tissues)**<br>• Provide the name of the Institutional Animal Care and Use Committee (IACUC) or other relevant ethics board that reviewed the study protocol, and indicate whether they approved this research or granted a formal waiver of ethical approval<br>• Include an approval number if one was obtained<br>• If the study involved *non-human primates*, add *additional details* about animal welfare and steps taken to ameliorate suffering<br>• If anesthesia, euthanasia, or any kind of animal sacrifice is part of the study, include briefly which substances and/or methods were applied<br><br>**Field Research**<br><br>Include the following details if this study involves the collection of plant, animal, or other materials from a natural setting:<br>• Field permit number<br>• Name of the institution or relevant body that granted permission	
**Data Availability**<br><br>Authors are required to make all data underlying the findings described fully available, without restriction, and from the time of publication. PLOS allows rare exceptions to address legal and ethical concerns. See the PLOS Data Policy and FAQ for detailed information.	Yes - all data are fully available without restriction

A Data Availability Statement describing where the data can be found is required at submission. Your answers to this question constitute the Data Availability Statement and **will be published in the article**, if accepted.<br><br>**Important:** Stating 'data available on request from the author' is not sufficient. If your data are only available upon request, select 'No' for the first question and explain your exceptional situation in the text box.<br><br>Do the authors confirm that all data underlying the findings described in their manuscript are fully available without restriction?	
**Describe where the data may be found in full sentences. If you are copying our sample text, replace any instances of XXX with the appropriate details.**<br><br>• If the data are **held or will be held in a public repository**, include URLs, accession numbers or DOIs. If this information will only be available after acceptance, indicate this by ticking the box below. For example: *All XXX files are available from the XXX database (accession number(s) XXX, XXX.).*<br>• If the data are all contained **within the manuscript and/or Supporting Information files**, enter the following: *All relevant data are within the manuscript and its Supporting Information files.*<br>• If neither of these applies but you are able to provide **details of access elsewhere**, with or without limitations, please do so. For example:<br><br>*Data cannot be shared publicly because of [XXX]. Data are available from the XXX Institutional Data Access / Ethics Committee (contact via XXX) for researchers who meet the criteria for access to confidential data.*<br><br>*The data underlying the results presented in the study are available from (include the name of the third party*	The raw sequence reads were deposited in the NCBI short read archive under the accession number: PRJNA655562

and contact information or URL).<br>• This text is appropriate if the data are owned by a third party and authors do not have permission to share the data.<br><br>* typeset	
Additional data availability information:	Tick here if the URLs/accession numbers/DOIs will be available only after acceptance of the manuscript for publication so that we can ensure their inclusion before publication.

Powered by Editorial Manager® and ProduXion Manager® from Aries Systems Corporation

Manuscript

Click here to access/download;Manuscript;PearmanPLOS_resub-v2.docx

Investigating variability in microbial community composition in replicate environmental DNA samples do[illegible] lake sediment cores

John K Pearman[1*], Georgia Thomson-Laing[1], Jamie D Howarth[2], Marcus J Vandergoes[3], Lucy Thompson[1], Andrew Rees[2], Susanna A Wood[1]

1. Coastal and Freshwater Group, Cawthron Institute, Private Bag 2, Nelson 7042, New Zealand
2. Victoria University of Wellington, PO Box 600, Wellington 6012, New Zealand
3. GNS Science, PO Box 30-368, Lower Hutt 5040, New Zealand.

* Corresponding author

Email: john.pearman@cawthron.org.nz

Abstract

Lake sediments are natural archives that accumulate information about biological communities and their surrounding catchments. Paleolimnology has traditionally focussed on identifying fossilized organisms to reconstruct past environments. In the last decade, the application of molecular methodologies has increased in paleolimnological studies, but further studies investigating factors such as sample heterogeneity and DNA degradation are required. In the present study we investigated bacterial community heterogeneity (16S rRNA metabarcoding) within depth slices. Sediment cores were collected from three lakes with differing sediment compositions. Samples were collected from a variety of depths (1-cm width) which represent a period of time of approximately 1,200 years. Triplicate samples were collected from each slice and bacterial 16S rRNA metabarcoding was undertaken on each sample. Rarefaction curves showed that except for the deepest (oldest) slices, the combination of three replicate samples were insufficient to characterise the entire bacterial diversity. However, shared Amplicon Sequence Variants (ASVs) accounted for the majority of the reads in each slice (max. shared proportional read abundance 96%, 86%, 65% in the three lakes). Non-metric multidimensional scaling analysis showed that replicates within a slice generally clustered together. A comparison of the variability among replicates showed that there was high community dissimilarity in older sediment in one of the cores, which was likely due to the laminae in the sediment core not been horizontal. Given that most paleolimnology studies explore broad scale shifts in community structure, rather than seeking to identify rare species, this study demonstrates that a single sample is adequate to characterise shifts in dominant bacterial ASVs.

Introduction

Understanding the lasting impacts of environmental decision making is fundamental to protecting and remediating Earth's biosphere. Long term insights are crucial to identifying and understanding ecological processes and inherent variability; however, biological monitoring rarely extends beyond a few decades [1]. Lake sediments accumulate information about autochthonous biological communities [2,3] and their surrounding catchments [4,5], enabling temporal insights into ecological processes [1]. Paleolimnology has traditionally focussed on identifying and enumerating organisms remains, but in the last decade, advances in molecular methodologies have permitted the identification of soft-bodied organisms that typically do not leave "footprints" in the sediment record [6 and references within].

The genetic material present in environmental samples, such as sediment, can be defined as environmental DNA (eDNA). This DNA originates from a complex mixture of living cells, unactive cells, fragments of dead cells and extracellular DNA from a variety of sources including faeces, urine and saliva [7]. Techniques such as metabarcoding, which enable the identification of taxa from samples containing a mix of species using high throughput sequencing and DNA reference databases, have recently been applied to characterise bacteria, Archaea, microbial eukaryotes, plants and vertebrates in lake sediments [8–12]. The application of these techniques has allowed new insights into temporal trends over decades, centuries and even millennium. Their use has the potential to allow testing of ecological theories which require long term data sets [e.g. evolutionary dynamics, anthropogenic impacts; ,6,13].

Before molecular techniques can be robustly applied to the analysis of biological communities in sediment cores, there are a number of methodological considerations that need to be addressed. Lake sediments have different compositions which can affect the absorption of DNA; for example, humic acids and clay materials have a stronger binding capacity for DNA than other sediments [14]. Mineralogy, porewater pH, and temperature can also impact DNA retention and degradation in sediments [15,16]. Reworking of sediments, and subsequent DNA transport, is known to occur through the movement of water, active growth of organisms and bioturbation [17]. However, initial research indicates that DNA leaching is minimal in saturated aquatic sediments [4,18].

Beyond complexities associated with sediment matrices, numerous studies, using metabarcoding approaches, have shown that DNA extraction efficiency and taxonomic

coverage of microbial species in soils is substantially different depending on the DNA extraction method applied [19–21]. A comprehensive review by Lear et al., [22] suggested that the most appropriate method for small sediment volumes was the DNeasy PowerSoil kit (Qiagen, Germany). While effort has gone into assessing methods-based variability in the terrestrial realm, little replication has been undertaken when extracting DNA from lake sediments.

An area of methodological consideration that requires further investigation is heterogeneity in eDNA across the small spatial scales of slices of the cores taken at different depths. Spatial heterogeneity within surface sediment samples has been previously shown [23,24] but heterogeneity within depth slices of sediment cores has received little attention. The investigation of variation within depth slices in sediment cores is especially vital as a large proportion of studies base their analysis on one or two DNA extractions from each depth slice [25–29]. In the present study, sediment cores were collected from three lakes with differing sediment compositions. The deepest sediment in the cores was estimated to be up to 1,200 years old. Triplicate DNA extractions followed by bacterial 16S rRNA metabarcoding were undertaken on samples collected from core slices (1 cm width) at depths ~~down the core~~ covering ~1200 years. We hypothesised that: (1) Replication would provide a better representation of the ASV richness within core slices and the richness would be better represen[illegible] with increasing depth; (2) community structure and composition will be more similar within slices than between slices; and (3) that there would be greater variability in the triplicates with increasing age of sediment due to differences in DNA degradation over time.

Methods

Sampling sites

The three lakes were chosen to cover a wide range of sediment types (Figure S1).

Lake Nganoke is a small (~3.1 ha), shallow (max. depth 2.3 m) lake located at 17 m above sea level (a.s.l) at the southern end of the Wairarapa Valley in the lower North Island of New Zealand (41°21′20″ S, 175°11′10″ E). The catchment of the lake (~174 ha) is incised into a terrace that has a maximum elevation of 100 m and is vegetated with high producing exotic grassland used for sheep and beef farming. The catchment lithology is characterised by early

Pleistocene river gravels and late Pleistocene beach deposits [30]. Sediments accumulating on the lake floor are highly organic silts formed largely from autochthonous sedimentation.

Lake Paringa is a medium sized lake (~475 ha) situated at 16 m a.s.l with a maximum depth of 58 m that has formed in an over-deepened glacial trough (~17,000 years ago). It is located on the West Coast of the South Island of New Zealand (43°43'10"S, 169°24'8"E), within a large catchment (~6,000 ha) that drains the ~1420 m a.s.l high frontal range of the Southern Alps. The catchment is primarily vegetated in undisturbed temperate podocarp rainforest [31]. The lithology of the lake catchment is predominantly cataclasites, mylonites and schists of the Rakaia Terrane east of the Alpine Fault and Greenland group metasediments to the west of the fault [32]. Lake Paringa's sedimentary fill is characterized by a repeating sequence of three deposits comprising (i) co-seismic megaturbidites formed by shaking-induced subaqueous mass wasting; (ii) post-seismic hyperpycnite stacks formed during periods of elevated fluvial sediment flux from earthquake-induced landsliding; and (iii) inter-seismic layered silts formed between earthquakes when the catchment is relatively geomorphically quiescent [31].

Lake Pounui is a small (~46 ha), shallow (max. depth 9.6 m), lowland coastal lake situated at 10 m a.s.l about 30 km northeast of Wellington, New Zealand (41°20'34"S, 175°6'48"E). Lake Pounui is thought to have formed ~3,000 years ago by the damming of a stream valley by beach sediment and alluvial gravels [33]. Lake Pounui's catchment (627 ha) extends to an elevation of 470 m in the foot hills of the Rimutaka Ranges. The majority of hillslopes are vegetated by unmodified indigenous beech-podocarp forest (96%), with the remainder in pastoral land cover [34]. The catchment lithology is composed on late Pleistocene marine benches underlying beach deposits, loess and alluvial gravels south east of the Wharekauhau Thrust fault and Esk Head sand and mudstones north west of the fault [30,33]; material eroded from these sources enters Lake Pounui [33,35,36]. The sediments accumulating on the lake floor are a mixture of allochthonous silts and very fine sands and autochthonous organic silts [33].

Sample collection

In lakes Pounui and Nganoke sediment cores were retrieved using a Uwitech (Mondsee, Austria) gravity corer with 2-m polyvinyl chloride (PVC; 65-mm dia.) barrels. In Lake Paringa a 6-m sediment core was collected using a Mackereth corer (50-mm dia.; [37]. Barrels were cleaned with 2% bleach prior to coring. The collected sediment cores were stored at 4°C in

darkness for up to 4 weeks until sub-sampling for lakes Nganoke and Paringa. The samples from Lake Pounui were collected and processed within 12 hours.

In the laboratory, the cores were sliced along the longitudinal plane, photographed, and described in detail including colour, texture and structure [38]. To prevent contamination, the top 2-3 mm of each half of the core was carefully removed with a sterile spatula. Slices of the cores were sampled at various depths from the top of the core (Supplementary Table 1 and Figure S2). Triplicate sub-samples (~ 0.5 g) were taken near the centre of the half-core using a sterile spatula and placed in separate tubes. Samples were kept frozen (-20°C) and in the dark until DNA extraction. Data on the age of sediment were sourced from other studies [31,39,40; Supplementary Table 1].

DNA Extraction, PCR and High Throughput Sequencing

Each step of the molecular analyses (i.e. DNA extraction, Polymerase Chain Reaction [PCR] set-up, template addition, PCR analysis) was conducted in dedicated separated sterile laboratories, with sequential workflow to ensure no cross-contamination. Rooms dedicated to DNA extraction, amplification set-up and template addition were equipped with ultra-violet (UV) sterilisation. UV sterilisation of the room and equipment was undertaken for 15 min before and after each use. The PCR set-up and template addition were always undertaken in laminar flow cabinets with HEPA filtration. Aerosol barrier tips were used throughout.

DNA was extracted from approximately 0.25 g of sediment using the DNeasy PowerSoil Kit (Qiagen, Germany) following the manufacturer's instructions on a QIAcube sample preparation robot (Qiagen). A negative extraction contro[illegible]as included every 23 samples.

The V3-V4 region of the bacterial 16S rRNA gene was amplified by PCR using the bacterial specific primers 341F: 5'-CCT ACG GGN GGC WGC AG-3' and 805R: 5'-GAC TAC HVG GGT ATC TAA TCC-3' [41]. The primers included Illumina^{TM} overhang adapters to allow dual indexing as described in Kozich et al., [42]. Triplicate PCR reactions were undertaken on each sample in 20-µL volumes. The reaction mixture consisted of 10 µL of 2 × PCR MyFi^{TM} Mix (Bioline), 1 µL of each primer (10 µM), 1.5 µL of bovine serum albumin (20 mg mL^{-1}) and 1.5 µL of template DNA. Cycling conditions were an initial 1 min 30 sec at 94°C before 30 cycles of 94°C for 30 s, 52°C for 30 s, 72°C for 45 s, with a final extension

step at 72°C for 5 min. Negative PCR controls were run alongside the samples. The triplicate PCR replicates were pooled and 20 µL was cleaned and normalized using SequalPrep Normalization plates (ThermoFisher Scientific, USA), resulting in a concentration of ~1 ng mL^{-1}. The cleaned samples were sent to Auckland Genomics Facility for paired-end (2 x 250 base pairs (bp)) sequencing on an Illumina Miseq™ platform. Sequence libraries were prepared as detailed in the Illumina 16S metagenomics library prep manual (https://support.illumina.com/documents/documentation/chemistry_documentation/16s/16s-metagenomic-library-prep-guide-15044223-b.pdf). One deviation from the protocol was that, after the indexing PCR, 5 µL of each sample was pooled, and clean-up was undertaken on the pooled library instead of samples being individually cleaned. The concentration and quality of the library was quantified using a bioanalyzer. The library was diluted to 4 nM and denatured, and a 15% PhiX spike-in was added. The library was further diluted to a final loading concentration of 7 ρM; raw sequence reads were deposited in the NCBI short read archive under the accession number: PRJNA655562

Primers were removed from the raw reads with cutadapt [43] allowing one mismatch. Sequences without primer sequences were discarded. Remaining sequences were processed with DADA2 [44] within the R framework [45]. Forward reads were truncated to 230 bp while reverse reads were truncated to 228 bp. The number of allowed maximum "expected errors" (maxEE) was two and four, to account for differences in sequence quality, for the forward and reverse respectively. The first 10^8 bp were used to construct a parametric error matrix. Sequences were dereplicated and amplicon sequence variants (ASVs) were inferred based on this error matrix. After, inference singletons were discarded and remaining paired-end reads were merged with a maximum of 1 bp mismatch and a required minimum overlap of 10 bp. Chimeric sequences were removed from the analysis using the removeBimeraDenovo function within DADA2.

The resulting ASVs were taxonomically classified in DADA2 using the rdp classifier [46] against the SILVA 132 database [47] with a bootstrap of 70. The results were combined into a phyloseq object [48] and any ASVs classified as eukaryotes, chloroplasts or mitochondria were removed. Negative controls were assessed and read numbers for ASVs found in the negative controls were removed from the samples via subtraction [49]. After this quality check step on

average greater than 99% of reads were retained in the lakes Nganoke and Pounui samples, and greater than 95% for Lake Paringa samples (Table S2).

Rarefactions curves were plotted showing the number of ASVs against sequencing depth for each replicate and for the replicates merged together using the R package ranacapa [50]. To compare samples within lakes, subsampling to an even sequencing depth was undertaken for each sample per lake (Lake Nganoke: 14,900 reads, Lake Paringa:18,800 reads, Lake Pounui: 9,800 reads). The number of observed ASVs was calculated per sample, using the phyloseq package [48] and plotted against depth to see changes in richness down the core. The number of shared ASVs and reads amongst the replicates was calculated to assess the similarity of the replicates within a slice. The community composition of each slice was assessed at the phyla level of taxonomic classification. Multivariate analysis, to see the clustering of replicates within slices, was undertaken on the samples using Bray Curtis dissimilarity matrices and visualized with non-metric multidimensional scaling (nMDS) plots. Each depth slice was assigned a number, increasing with depth, to assist with visualization of data. Smaller numbers represent the top of the core and larger numbers deeper slices in the core. Exact depths/ages are detailed in Supplementary Table 1. Comparisons of the mean similarity (1- Bray Curtis and 1- Jaccard) within and between depth slices were calculated using a Kruskal-Wallis test in R. The mean similarity (1- Bray Curtis) of the triplicates for each slice was calculated and plotted with a linear regression calculated to assess differences in similarity with depth. Figures were produced in R using the package ggplot2 [51].

Results and Discussion

The use of molecular techniques to characterise biological communities in environmental samples has been increasing over the past decade. There is an ongoing need to ensure an in-depth knowledge of the robustness and caveats of these techniques for specific applications. A total of 5,317,829, 1,880,055 and 1,827,617 sequences were obtained for sediment core samples from lakes Nganoke, Paringa and Pounui, respectively. After bioinformatically subsampling to an even depth, this resulted in 45,139 ASVs for Lake Nganoke, 19,540 ASVs for Lake Paringa, and 51,202 ASVs for Lake Pounui.

Metabarcoding samples often contain a high number of low abundance ASVs [52,53] and large sequencing efforts are required to reveal the full level of diversity. This was observed in the

present study and is highlighted by the accumulation curves which in general did not reach a plateau, especially in samples near the top of the core and for the merged replicates (Figure 1 and Figure S3-5). Similar results have been observed in other lake sediment studies that have used the same (DADA2) bioinformatic pipelines [54]. These pipelines have a finer scale of resolution than previous operational taxonomic unit methods [44]. It is however feasible that a portion of the diversity observed could be due to PCR/sequencing errors that were not been detected in the bioinformatics pipeline. The accumulation curves in the present study suggest that taking three replicate samples is insufficient to capture the full extent of bacterial diversity in most sediment core slices, although a fuller inventory of the ASVs in deeper samples was achieved (Figure 1).

The more complete inventory of ASVs in the deeper sections of the core could be due to the lower ASV richness observed with depth in the core (Figure S6). This trend could be the result of a couple of factors. Firstly, at deeper depths there may be increased damage to the DNA present in the sediment through strand breakage, abasic sites, miscoding lesions and crosslinks [55,56]. This can result in fewer amplifiable templates and as the majority of the ASVs present in metabarcoding samples are rare and of low abundance, it is likely that a proportion of these would no longer be detected [6]. It should be noted that the rates of DNA degradation are affected by a combination of abiotic (e.g. mineralogical composition, organic load, temperature and stable stratification) and biotic factors (e.g. degradation via DNase activity) and thus the effect of DNA degradation is likely to vary between lakes [6,57]. Secondly, the samples taken in this study cover a time scale ranging from ~800 years (Pounui) to ~1200 years (Paringa) before present. Thus, temporal changes in the ecology of the lakes, affected by pressures such as climate change and anthropogenic impacts, could have led to a more diverse bacterial community in more recent times. An in-depth analysis of the composition of the changes in microbial communities is the subject of other research, but a high level analysis shows temporal changes. For example, the phyla Bacteroidetes increases in relative abundance at the top of the core in all three lakes (Figure S7). Other phyla, such as Cyanobacteria (Pounui) and Nitrospirae (Nganoke) also increase in relative abundance in recent times indicating shifts in lake dynamics. A combination of methods could be applied to get a better indication of sediment core richness including increasing sequencing depth within a sample, undertaking multiple DNA extractions at a depth, or increasing the number of PCR replicates undertaken [28,58].

The number of ASVs shared amongst the three replicates was lowest in Lake Pounui (6.5-12.2%), with Lake Paringa (11.7-25.3%) and Lake Nganoke (13.7-23.4%) having higher proportions of shared ASVs. There was no significant trend in the number of shared ASVs with depth ($p > 0.05$; Figure S8 A-C). Although the number of ASVs shared amongst the three replicates was relatively low, the majority of reads were accounted for by shared ASVs. In Lake Paringa, shared ASVs accounted for a maximum of 96% (range 76-96%) of the reads within a slice, compared to 69-86% in Lake Nganoke and 45-65% in Lake Pounui. Bioturbation, namely organism-induced mixing of sediment in both vertical and horizontal directions, might affect the numbers of shared reads within each lake. Lakes Nganoke and Pounui both have kākahi (freshwater mussel; *Echyridella menziesi*) which would likely result in higher bioturbation rates than in Lake Paringa. With lower levels of bioturbation, the stratigraphy would be less disturbed leading to more homogeneous replicates at any depth and consequently a higher shared number of reads. While lakes Nganoke and Pounui have higher levels of bioturbation, likely reducing the number of shared reads amongst triplicates, there is lower sedimentary layering in Lake Nganoke. Bioturbation is therefore likely to be mixing similar layers and distinct changes in the community would be reduced.

Similarity comparisons were undertaken to further explore differences within and between depth slices. The mean similarity of pairwise comparisons within slices (based on relative abundance data) was found to be significantly higher than the mean of pair wise similarity comparisons between slices (1 – Bray Curtis; Nganoke: $p < 0.001$; Paringa: $p < 0.001$; Pounui: $p < 0.001$; Figure 2A-C). A similar trend was noted when only community structure was assessed (i.e., presence/absence data; 1 – Jaccard; Nganoke: $p < 0.001$; Paringa: $p < 0.001$; Pounui: $p < 0.001$; Figure 2D-F). This indicates that the variability in bacteria communities within a depth slice is lower than amongst depths. We anticipated that there would be marked differences in bacterial community composition and structure across depths in all lakes. Over the last ~1,000 years these lakes have been subjected to multiple natural and anthropogenic disturbances which would have impacted their microbial communities (Howarth et al., 2012; Parish, 2020, Andrew Rees, Victoria University unpub. data). Studies on these taxonomic shifts and their potential drivers are the focus of ongoing studies.

The non-metric multidimensional (NMDS) plots showed that depth slice replicates generally clustered together (Figure 3). There were several notable exceptions, for example, triplicates were relatively widely dispersed in Lake Paringa slices 9-11. Reasons for the lower

homogeneity in these samples is unknown, and technical origins of this variation cannot be ruled out, but there could be greater sediment heterogeneity due to rapid changes in the landscape around the lakes (caused by impacts of human settlement or natural disturbance, i.e., earthquakes). In Lake Pounui slices 11, 19 and 20 (Figure 3) had a wider spread. Slices 19 and 20 correspond to a region of the core where the laminae are not horizontal and have dip of 20-30 degrees (Supplementary Figure S2). Replicates taken in this part of the core may be sampling across the stratigraphy which would consequently lower the similarity and may explain the lack of clustering. In Lake Nganoke, one of the replicates from slice 6 was very similar in composition to the slice above. Reworking of sediment by living organisms has been documented and could account for this observation although there was 5 cm between these layers [17].

Sediment from a range of depths/ages was analysed to determine whether there was greater variability (lower similarity between triplicates) in older sediment. We hypothesised that greater variability would occur in older sediment and that this might be caused by stochastic differences in DNA degradation. However, this hypothesis was not supported in lakes Nganoke and Paringa, although in Lake Paringa there was a significant positive relationship between similarity and slice depth ($p = 0.0241$, $r^2 = 0.357$; Figure 4). Lake Paringa, ~~is~~ the deepest core and covers the longest temporal period of the lakes studied here and a significant increase in the proportion of shared reads with depth is observed ($p < 0.001$, $r^2 = 0.658$; Figure S8E). This could indicate that DNA degradation in the core is removing the rarer low abundance ASVs and that these deeper slices are thus dominated by abundant ASVs that are shared amongst all replicates. Further investigation with other longer cores covering a longer time period is required. Only Lake Pounui showed a significant decline in similarity within a slice with depth ($p = 0.0196$, $r^2 = 0.233$; Figure 4). The decrease in similarity at these depths is most likely to be due to the non-horizontal laminae deeper in the core, which would likely result in the replicates representing different time periods.. If laminae are not horizontal then increased variability in replication may be observed and results would need to be carefully considered as replicates may represent different temporal periods.

Conclusion

In summary, the results of this study indicate that the analysis of triplicate samples is insufficient to capture the full bacterial diversity in the slice of sediment cores. However, most

paleolimnology studies do not aim to identify rare species, but rather explore broad scale shifts in community structure [6,26]. There may be exceptions to this when investigating rare or invasive species [59], and in these instances it might be more appropriate to use targeted methods such as droplet digital PCR~~,~~ or increase sequencing depth and refrain from rarefaction, a technique which has been recommended for marine biosecurity surveillance [60]. The percentage of reads accounted for by shared ASVs amongst replicates was high, especially in lakes Nganoke and Paringa, demonstrating that individual replicates contain the dominant bacterial ASV's. In general, there was strong clustering of triplicates in the NMDS, and triplicates within a slice were distinct from those in other slices; however, there were exceptions where one replicate was markedly different. Reasons for this are unknown but could be related to bioturbation or sediment heterogeneity. This highlights the need for caution when interpreting these types of data. When dramatic shifts are observed, we recommend analysis of further replicates and increased sampling in layers directly above and below to ascertain when the shift in the community occurred. Critically, major community shifts need to be interpreted hand-in-hand with stratigraphic data. No conclusive trend was observed when looking at the similarity of the community of the three replicates with depth. Only Lake Pounui displayed a significant negative trend with a decrease in similarity with depth and this was most likely related to laminae not being horizontal at these depths. Overall, we show that the variation within a depth slice is lower than amongst slices giving confidence to the use of eDNA methodologies to assessing microbial communities within sediment cores.

Acknowledgements

This research was funded by the New Zealand Ministry of Business, Innovation and Employment research programme - Our lakes' health; past, present and future (C05X1707). The authors thank Laura Biessy (Cawthron Institute), Lizette Reyes (GNS Science) and Jake Parrish (Victoria University) for field and laboratory assistance. Ngāti Kahungunu ki Wairarapa (lakes Nganoke and Pounui) and Te Runanga o Makaawhio (Lake Paringa) are acknowledged for their support of this project. The landowners at lakes Nganoke and Pounui are thanked for access to the launching sites. The Department of Conservation is acknowledged for assistance with permitting (Lake Paringa).

References

1. Seddon AWR, Mackay AW, Baker AG, Birks HJB, Breman E, Buck CE, et al. Looking forward through the past: identification of 50 priority research questions in palaeoecology. J Ecol. 2014;102: 256–267.

2. Capo E, Debroas D, Arnaud F, Domaizon I. Is planktonic diversity well recorded in sedimentary DNA? Toward the reconstruction of past protistan diversity. Microb Ecol. 2015;70: 865–875. doi:10.1007/s00248-015-0627-2

3. Millet L, Arnaud F, Heiri O, Magny M, Verneaux V, Desmet M. Late-Holocene summer temperature reconstruction from chironomid assemblages of Lake Anterne, northern French Alps: The Holocene. 2009 [cited 2 Apr 2020]. doi:10.1177/0959683608100576

4. Giguet-Covex C, Pansu J, Arnaud F, Rey P-J, Griggo C, Gielly L, et al. Long livestock farming history and human landscape shaping revealed by lake sediment DNA. Nat Commun. 2014;5: 1–7. doi:10.1038/ncomms4211

5. Pansu J, Giguet-Covex C, Ficetola GF, Gielly L, Boyer F, Zinger L, et al. Reconstructing long-term human impacts on plant communities: an ecological approach based on lake sediment DNA. Mol Ecol. 2015;24: 1485–1498. doi:10.1111/mec.13136

6. Domaizon I, Winegardner A, Capo E, Gauthier J, Gregory-Evans I. DNA-based methods in paleolimnology: new opportunities for investigating long-term dynamics of lacustrine biodiversity. J Paleolimnol. 2017;58: 1–21.

7. Taberlet P, Bonin A, Zinger L, Coissac E. Environmental DNA: For Biodiversity Research and Monitoring. Oxford University Press; 2018.

8. Alsos IG, Sjögren P, Edwards ME, Landvik JY, Gielly L, Forwick M, et al. Sedimentary ancient DNA from Lake Skartjørna, Svalbard: Assessing the resilience of arctic flora to Holocene climate change. 2016. doi:10.1177/0959683615612563

9. Kisand V, Talas L, Kisand A, Stivrins N, Reitalu T, Alliksaar T, et al. From microbial eukaryotes to metazoan vertebrates: Wide spectrum paleo-diversity in sedimentary ancient DNA over the last 14,500 years. Geobiology. 2018;16: 628–639. doi:10.1111/gbi.12307

10. Parducci L, Bennett KD, Ficetola GF, Alsos IG, Suyama Y, Wood JR, et al. Ancient plant DNA in lake sediments. New Phytol. 2017;214: 924–942. doi:10.1111/nph.14470

11. Poulain AJ, Aris-Brosou S, Blais JM, Brazeau M, Keller W (Bill), Paterson AM. Microbial DNA records historical delivery of anthropogenic mercury. ISME J. 2015;9: 2541–2550. doi:10.1038/ismej.2015.86

12. Vuillemin A, Ariztegui D, Leavitt PR, Bunting L. Recording of climate and diagenesis through sedimentary DNA and fossil pigments at Laguna Potrok Aike, Argentina. Biogeosciences. 2016;13: 2475–2492. doi:10.5194/bg-13-2475-2016

13. Bálint M, Pfenninger M, Grossart H-P, Taberlet P, Vellend M, Leibold MA, et al. Environmental DNA time series in ecology. Trends Ecol Evol. 2018;33: 945–957. doi:10.1016/j.tree.2018.09.003

14. Dell'Anno A, Stefano B, Danovaro R. Quantification, base composition, and fate of extracellular DNA in marine sediments. Limnol Oceanogr. 2002;47: 899–905. doi:10.4319/lo.2002.47.3.0899

15. Levy-Booth DJ, Campbell RG, Gulden RH, Hart MM, Powell JR, Klironomos JN, et al. Cycling of extracellular DNA in the soil environment. Soil Biol Biochem. 2007;39: 2977–2991. doi:10.1016/j.soilbio.2007.06.020

16. Torti A, Lever MA, Jørgensen BB. Origin, dynamics, and implications of extracellular DNA pools in marine sediments. Mar Genomics. 2015;24: 185–196. doi:10.1016/j.margen.2015.08.007

17. Rawlence NJ, Lowe DJ, Wood JR, Young JM, Churchman GJ, Huang Y-T, et al. Using palaeoenvironmental DNA to reconstruct past environments: progress and prospects. J Quat Sci. 2014;29: 610–626. doi:10.1002/jqs.2740

18. Anderson-Carpenter LL, McLachlan JS, Jackson ST, Kuch M, Lumibao CY, Poinar HN. Ancient DNA from lake sediments: Bridging the gap between paleoecology and genetics. BMC Evol Biol. 2011;11: 30. doi:10.1186/1471-2148-11-30

19. Holmsgaard PN, Norman A, Hede SChr, Poulsen PHB, Al-Soud WA, Hansen LH, et al. Bias in bacterial diversity as a result of Nycodenz extraction from bulk soil. Soil Biol Biochem. 2011;43: 2152–2159. doi:10.1016/j.soilbio.2011.06.019

20. Knauth S, Schmidt H, Tippkötter R. Comparison of commercial kits for the extraction of DNA from paddy soils. Lett Appl Microbiol. 2013;56: 222–228. doi:10.1111/lam.12038

21. Young JM, Rawlence NJ, Weyrich LS, Cooper A. Limitations and recommendations for successful DNA extraction from forensic soil samples: A review. Sci Justice. 2014;54: 238–244. doi:10.1016/j.scijus.2014.02.006

22. Lear G, Dickie I, Banks J, Boyer S, Buckley H, Buckley T, et al. Methods for the extraction, storage, amplification and sequencing of DNA from environmental samples. N Z J Ecol. 2018;42: 10. doi:10.20417/nzjecol.42.9

23. Brannock PM, Halanych KM. Meiofaunal community analysis by high-throughput sequencing: Comparison of extraction, quality filtering, and clustering methods. Mar Genomics. 2015;23: 67–75. doi:10.1016/j.margen.2015.05.007

24. Lejzerowicz F, Esling P, Pawlowski J. Patchiness of deep-sea benthic Foraminifera across the Southern Ocean: Insights from high-throughput DNA sequencing. Deep Sea Res Part II Top Stud Oceanogr. 2014;108: 17–26. doi:10.1016/j.dsr2.2014.07.018

25. Monchamp M-E, Spaak P, Domaizon I, Dubois N, Bouffard D, Pomati F. Homogenization of lake cyanobacterial communities over a century of climate change and eutrophication. Nat Ecol Evol. 2018;2: 317–324. doi:10.1038/s41559-017-0407-0

26. Capo E, Debroas D, Arnaud F, Perga M-E, Chardon C, Domaizon I. Tracking a century of changes in microbial eukaryotic diversity in lakes driven by nutrient enrichment and climate warming. Environ Microbiol. 2017;19: 2873–2892. doi:10.1111/1462-2920.13815

27. Monchamp M-E, Walser J-C, Pomati F, Spaak P. Sedimentary DNA Reveals Cyanobacterial Community Diversity over 200 Years in Two Perialpine Lakes. Appl Environ Microbiol. 2016;82: 6472–6482. doi:10.1128/AEM.02174-16

28. Ficetola GF, Pansu J, Bonin A, Coissac E, Giguet-Covex C, De Barba M, et al. Replication levels, false presences and the estimation of the presence/absence from eDNA metabarcoding data. Mol Ecol Resour. 2015;15: 543–556. doi:10.1111/1755-0998.12338

29. Parducci L, Väliranta M, Salonen JS, Ronkainen T, Matetovici I, Fontana SL, et al. Proxy comparison in ancient peat sediments: pollen, macrofossil and plant DNA. Philos Trans R Soc Lond B Biol Sci. 2015;370: 20130382. doi:10.1098/rstb.2013.0382

30. Begg JG, Johnston MR. Geology of the Wellington area 1:250 000 geological map 10. Institute of Geological and Nuclear Sciences; 2000. Available: https://natlib.govt.nz/records/22189402

31. Howarth JD, Fitzsimons SJ, Norris RJ, Jacobsen GE. Lake sediments record cycles of sediment flux driven by large earthquakes on the Alpine fault, New Zealand. Geology. 2012;40: 1091–1094. doi:10.1130/G33486.1

32. Rattenbury M, Jongens R, Cox S. Geology of the Haast Area 1:250 000. Geological Map 14. Institute of Geological & Nuclear Sciences; 2010.

33. Turner GM, Lillis DA. A palaeomagnetic secular variation record for New Zealand during the past 2500 years. Phys Earth Planet Inter. 1994;83: 265–282. doi:10.1016/0031-9201(94)90093-0

34. Perrie A, Milne JR. Lake water quality and ecology in the Wellington region: State and trends. Wellington, NZ: Greater Wellington Regional Council; 2012. Report No.: GW/EMI-T-12/139.

35. Mcfadgen BG. Archaeology of the Wellington Conservancy: Wairarapa. A study in tectonic archaeology. Wellington, New Zealand: Department of Conservation; 2003. Available: https://www.semanticscholar.org/paper/Archaeology-of-the-Wellington-Conservancy%3A-A-study-Mcfadgen/73b4da315cbcb3c6f95add49b34fd9d9aeadca8c

36. Palmer AS, Vucetich CG, McGlone M, Harper MA. Last Glacial loess and early Last Glacial vegetation history of Wairarapa Valley, New Zealand. N Z J Geol Geophys. 1989;32: 499–513. doi:10.1080/00288306.1989.10427557

37. Mackereth FJH. A portable core sampler for lake deposits. Limnol Oceanogr. 1958;3: 181–191. doi:10.4319/lo.1958.3.2.0181

38. Schnurrenberger D, Russell J, Kelts K. Classification of lacustrine sediments based on sedimentary components. J Paleolimnol. 2003;29: 141.

39. Cochrane L. Reconstructing Ecological Change, Catchment Disturbance, and Anthropogenic Impact over the last 3000 years at Lake Pounui, Wairarapa, New Zealand. Victoria University, Wellington. 2017. Available: http://researcharchive.vuw.ac.nz/handle/10063/6665

40. Parish J. Reconstructing the effects of riparian planting in mitigating agriculturally intensified run off from paleo lake records: A case study of Lake Nganoke. MSc thesis unpublished, Victoria University, Wellington. 2020.

41. Klindworth A, Pruesse E, Schweer T, Peplies J, Quast C, Horn M, et al. Evaluation of general 16S ribosomal RNA gene PCR primers for classical and next-generation sequencing-based diversity studies. Nucleic Acids Res. 2013;41. doi:10.1093/nar/gks808

42. Kozich JJ, Westcott SL, Baxter NT, Highlander SK, Schloss PD. Development of a dual-index sequencing strategy and curation pipeline for analyzing amplicon sequence data on the MiSeq Illumina sequencing platform. Appl Environ Microbiol. 2013;79: 5112–5120. doi:10.1128/AEM.01043-13

43. Martin M. Cutadapt removes adapter sequences from high-throughput sequencing reads. EMBnet.journal. 2011;17: 10–12. doi:10.14806/ej.17.1.200

44. Callahan BJ, McMurdie PJ, Rosen MJ, Han AW, Johnson AJA, Holmes SP. DADA2: high-resolution sample inference from Illumina amplicon data. Nat Methods. 2016;13: 581.

45. Team RC. R: A language and environment for statistical computing. Vienna, Austria: R Foundation for Statistical Computing; 2014. 2020.

46. Wang Q, Garrity GM, Tiedje JM, Cole JR. Naive Bayesian classifier for rapid assignment of rRNA sequences into the new bacterial taxonomy. Appl Environ Microbiol. 2007;73: 5261–5267.

47. Pruesse E, Quast C, Knittel K, Fuchs BM, Ludwig W, Peplies J, et al. SILVA: a comprehensive online resource for quality checked and aligned ribosomal RNA sequence data compatible with ARB. Nucleic Acids Res. 2007;35: 7188–7196. doi:10.1093/nar/gkm864

48. McMurdie PJ, Holmes S. phyloseq: An R package for reproducible interactive analysis and graphics of microbiome census data. PloS One. 2013;8: e61217. doi:10.1371/journal.pone.0061217

49. Bell KL, Burgess KS, Botsch JC, Dobbs EK, Read TD, Brosi BJ. Quantitative and qualitative assessment of pollen DNA metabarcoding using constructed species mixtures. Mol Ecol. 2019;28: 431–455. doi:10.1111/mec.14840

50. Kandlikar GS, Gold ZJ, Cowen MC, Meyer RS, Freise AC, Kraft NJB, et al. ranacapa: An R package and Shiny web app to explore environmental DNA data with exploratory statistics and interactive visualizations. F1000Research. 2018;7. doi:10.12688/f1000research.16680.1

51. Wickham H. ggplot2: elegant graphics for data analysis. Springer; 2016.

52. Lynch MD, Neufeld JD. Ecology and exploration of the rare biosphere. Nat Rev Microbiol. 2015;13: 217.

53. Pedros-Alio C. The rare bacterial biosphere. Annu Rev Mar Sci. 2012;4: 449–66. doi:10.1146/annurev-marine-120710-100948

54. Pearman JK, Biessy L, Thomson-Laing G, Waters S, Vandergoes MJ, Howarth JD, et al. Local factors drive bacterial and microeukaryotic community composition in lake surface sediment collected across an altitudinal gradient. FEMS Microbiol Ecol. 2020;96. doi:10.1093/femsec/fiaa070

55. Hofreiter M, Serre D, Poinar HN, Kuch M, Pääbo S. Ancient DNA. Nat Rev Genet. 2001;2: 353–359. doi:10.1038/35072071

56. Lindahl T. Instability and decay of the primary structure of DNA. Nature. 1993;362: 709–715. doi:10.1038/362709a0

57. Corinaldesi C, Beolchini F, Dell'Anno A. Damage and degradation rates of extracellular DNA in marine sediments: implications for the preservation of gene sequences. Mol Ecol. 2008;17: 3939–3951.

58. Lanzén A, Lekang K, Jonassen I, Thompson EM, Troedsson C. DNA extraction replicates improve diversity and compositional dissimilarity in metabarcoding of eukaryotes in marine sediments. PLoS ONE. 2017;12. doi:10.1371/journal.pone.0179443

59. Savichtcheva O, Debroas D, Kurmayer R, Villar C, Jenny JP, Arnaud F, et al. Quantitative PCR Enumeration of Total/Toxic Planktothrix rubescens and Total Cyanobacteria in Preserved DNA Isolated from Lake Sediments. Appl Environ Microbiol. 2011;77: 8744–8753. doi:10.1128/AEM.06106-11

60. von Ammon U, Wood SA, Laroche O, Zaiko A, Tait L, Lavery S, et al. Combining morpho-taxonomy and metabarcoding enhances the detection of non-indigenous marine pests in biofouling communities. Sci Rep. 2018;8: 1–11. doi:10.1038/s41598-018-34541-1

Figure legends

Fig. 1. Rarefaction curves displaying the observed number of Amplicon Sequence Variants (ASVs) with increasing number of reads for four distinct slices (top of core, 2 slices at intermediate core depth and bottom of core (see Table S1 for depths of slices); plots for all slices are in Supplementary Figures 3-5) representative of distinct depth slices in sediment core samples taken from; A) Lake Nganoke, B) Lake Paringa, and C) Lake Pounui. Curves are depicted for individual replicates and as a combination of replicates (Merged). The level of sub sampling to an even depth used in the further analysis was indicated by the dashed vertical line.

Fig. 2. Similarity comparisons (1 – Bray Curtis) within and between depth slices for Lake Nganoke (A), Lake Paringa (B), and Lake Pounui (C). Similarity comparisons (1 – Jaccard) within and between depth slices for Lake Nganoke (D), Lake Paringa (E), and Lake Pounui (F). Boxes display the first and third quartile spread of the data, with the line in the box indicating the median, the whiskers denoting the minimum and maximum values and the dots as outliers of the data.

Fig. 3. Non-metric multidimensional scaling plot based on Bray Curtis distance matrices for samples taken from Lake Nganoke (A), Lake Paringa (B), and Lake Pounui (C). Points are coloured by depth slice. Approximate ages of slices vary between lakes and are given in Supplementary Table 1. Stress values for the NMDS plots were Nganoke: 0.048; Paringa: 0.066 and Pounui: 0.055.

Fig. 4. Linear regression of similarity (1-Bray Curtis) and slice in Lake Nganoke (A), Lake Paringa (B), and Lake Pounui (C). Slices are organised by depth slice (1 being the most recent). Approximate ages of slices vary between lakes and are given in Supplementary Table 1.

Supplementary Info

Table S1: Sub-bottom depth and age of each slice for lakes Nganoke, Paringa and Pounui.

Table S2: Number of reads before and after removing contaminating sequences from controls by subtraction.

Figure S1: Map of the position of the lakes.

Figure S2: Photographs of the sediment cores sampled with the sampling slices depicted.

Figure S3: Rarefaction curves displaying the observed number of Amplicon Sequence Variants (ASVs) with increasing number of reads for four distinct slices representative of distinct depth ~~slice~~ (see Table S1 for depths of slices) in sediment core samples taken from Lake Nganoke.

Figure S4: Rarefaction curves displaying the observed number of Amplicon Sequence Variants (ASVs) with increasing number of reads for four distinct slices representative of distinct depth slice (see Table S1 for depths of slices) in sediment core samples taken from Lake Paringa.

Figure S5: Rarefaction curves displaying the observed number of Amplicon Sequence Variants (ASVs) with increasing number of reads for four distinct slices representative of distinct depth slice (see Table S1 for depths of slices) in sediment core samples taken from Lake Pounui.

Figure S6: The total richness per slice (triplicates combined) with depth down the core. A - Lake Nganoke; B – Lake Paringa; C- Lake Pounui.

Figure S7: Relative proportional composition of the community at phyla level with depth down the core. A - Lake Nganoke; B – Lake Paringa; C- Lake Pounui. Phyla that did not account for on average > 1% of the community were not show so bars do not add up to 100%.

Figure S8: The proportion of shared ASVs (A – Lake Nganoke; B – Lake Paringa; C - Lake Pounui) and reads (D – Lake Nganoke; E – Lake Paringa; F - Lake Pounui) against depth. Note the different scales.

Figure 1

Click here to access/download;Figure;Figure1.eps

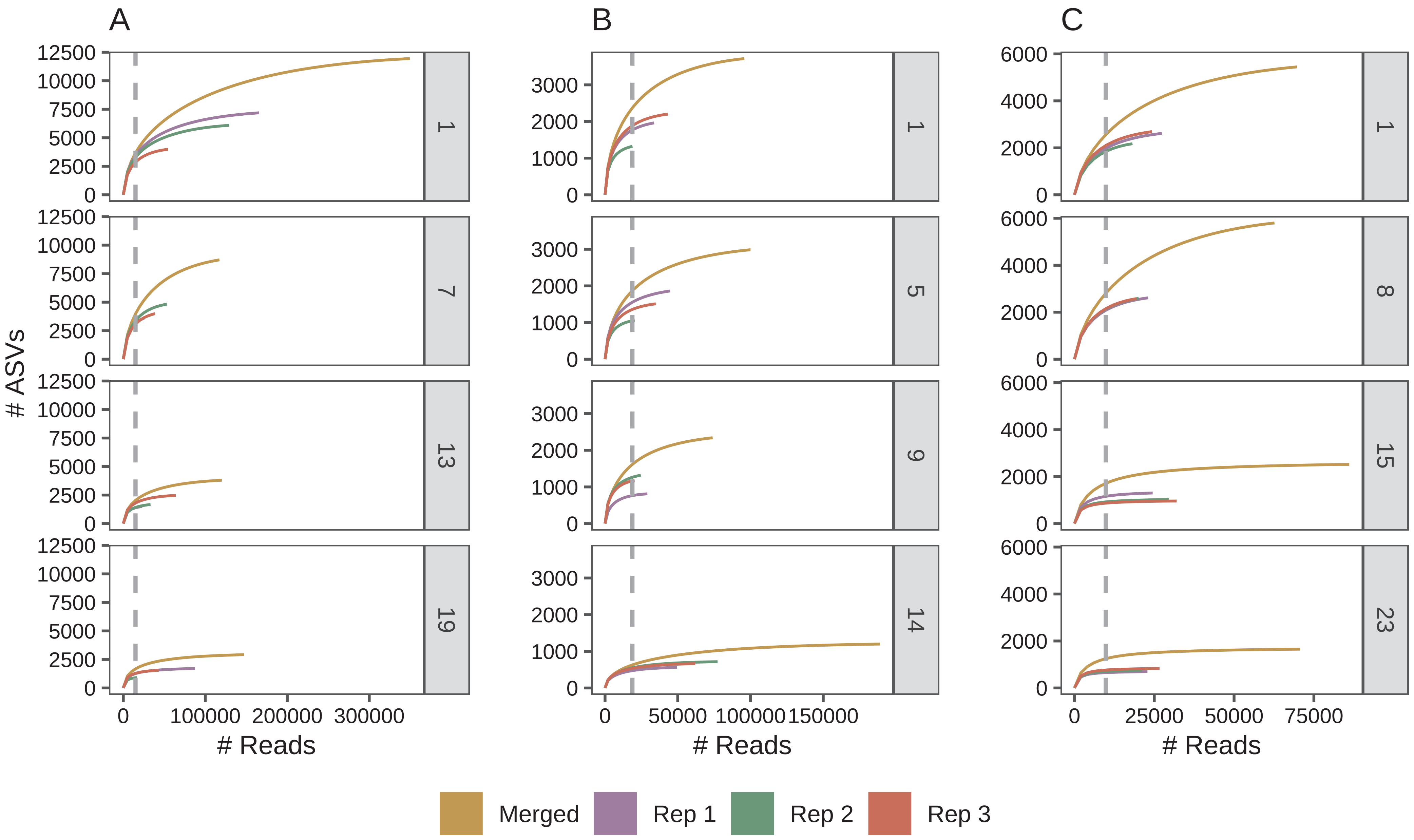

Figure 2

Click here to access/download;Figure;Figure2.eps

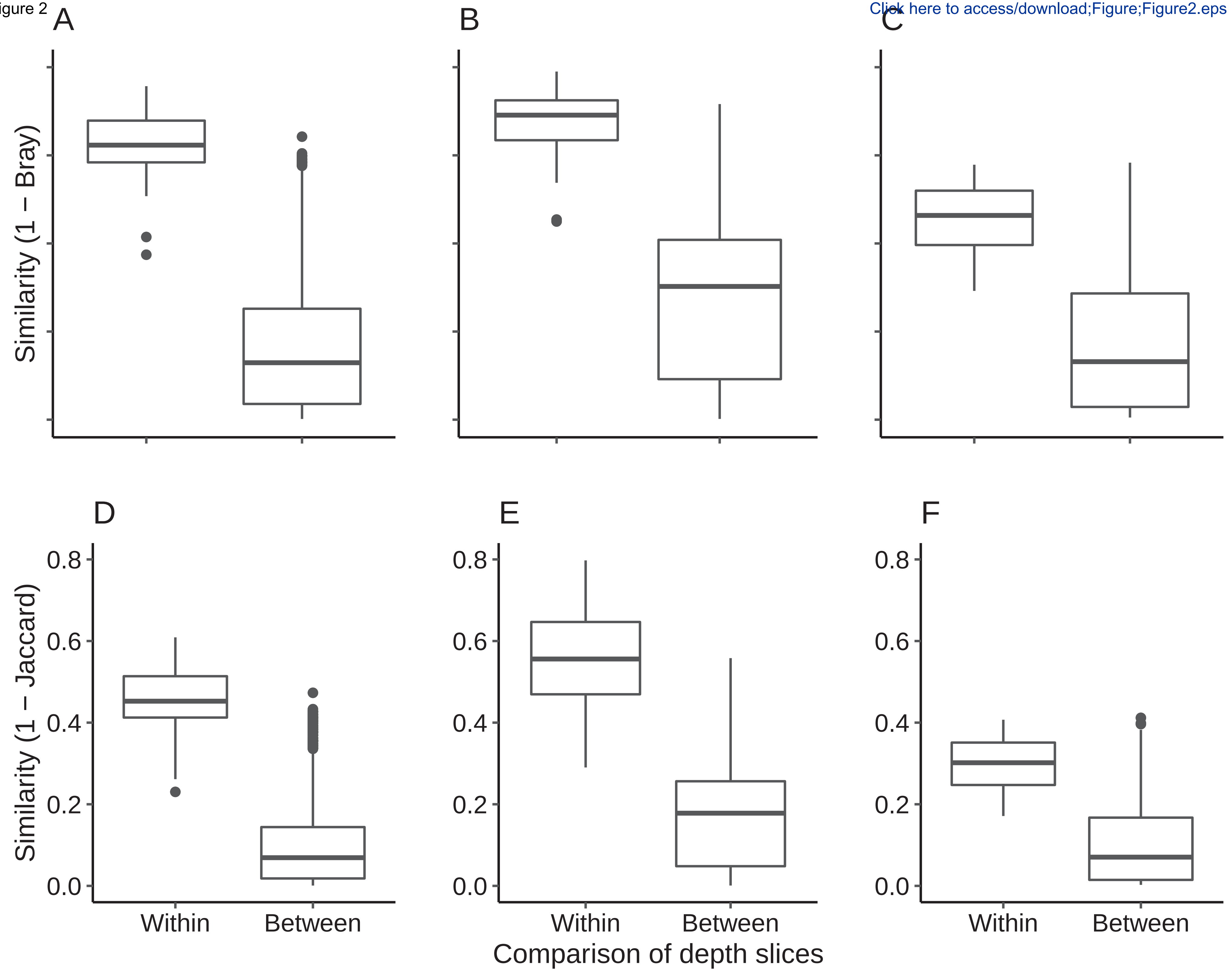

Figure 3

Click here to access/download;Figure;Figure3.eps

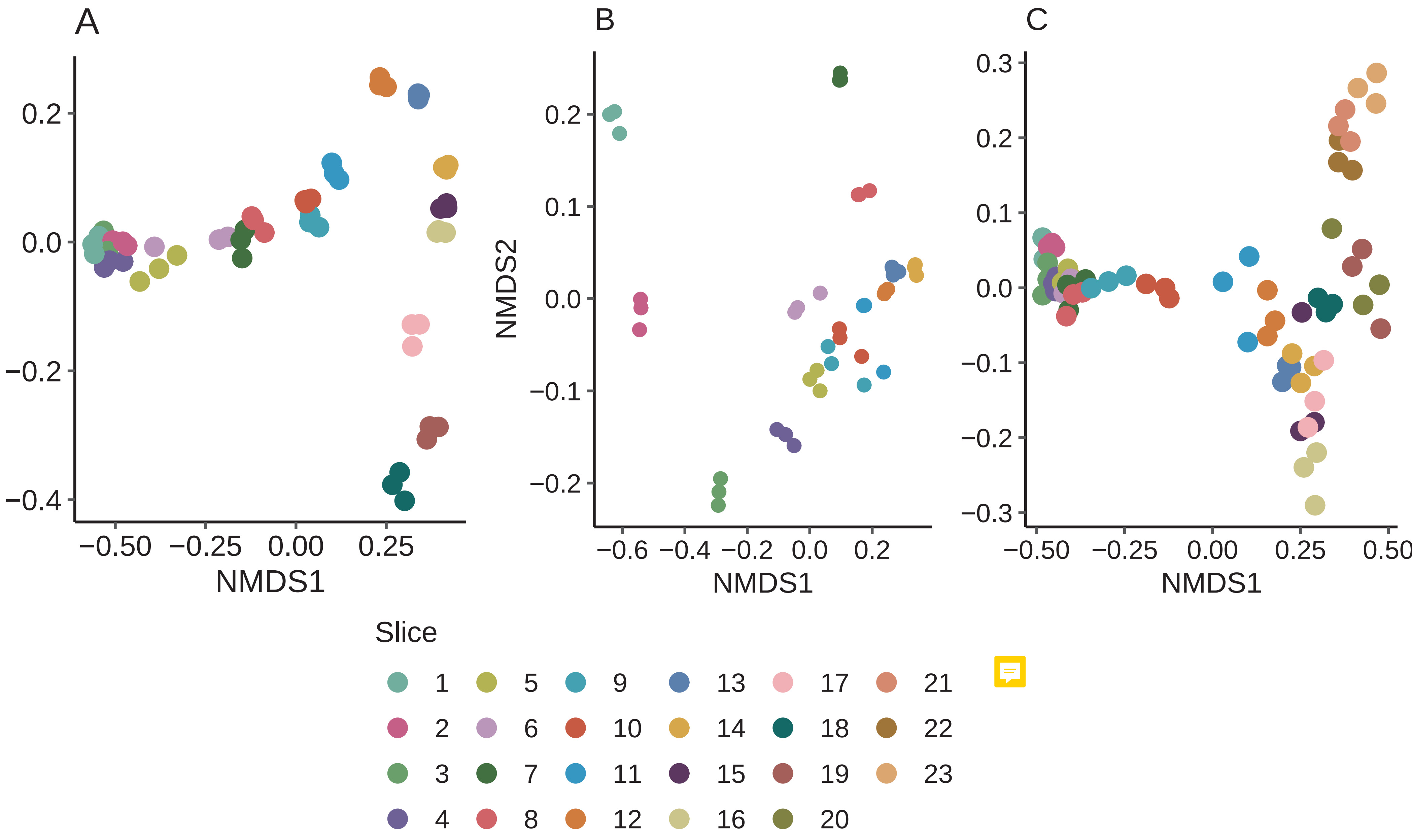

Figure 4

Click here to access/download;Figure;Figure4.eps

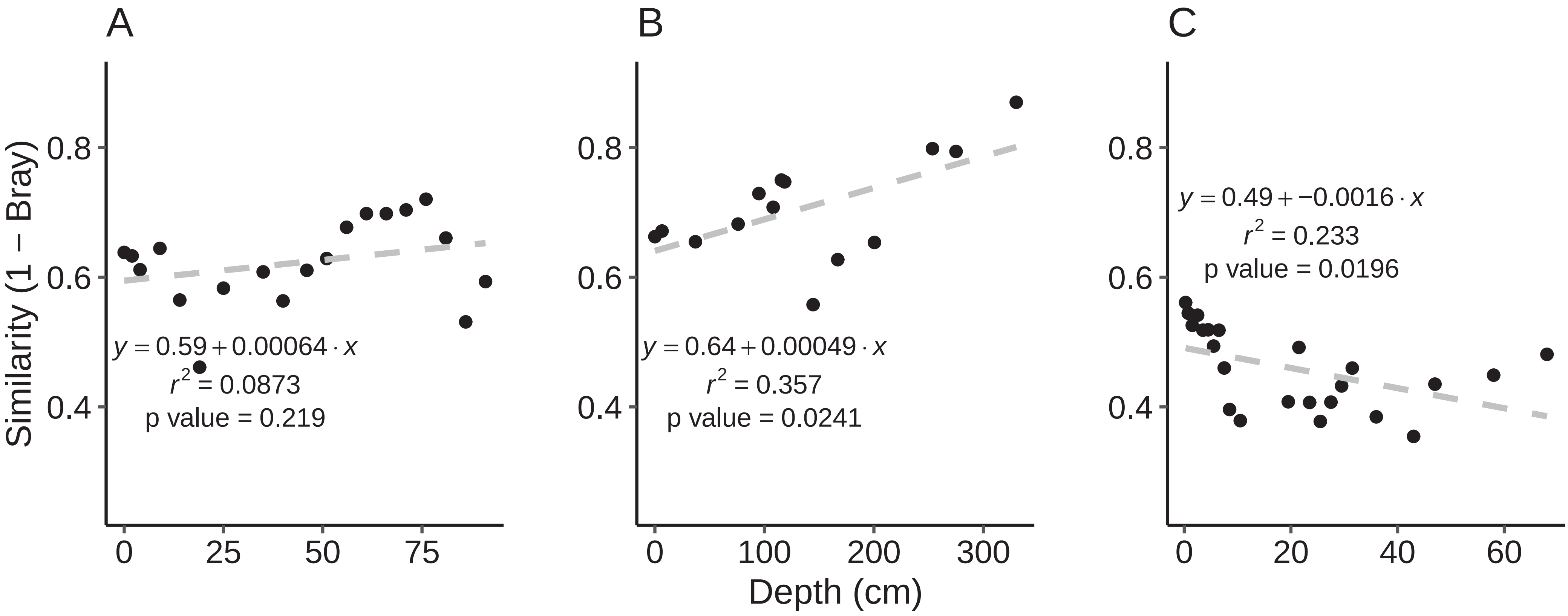

Supporting Information Table 1

Click here to access/download
Supporting Information
TableS1.xlsx

Supporting Information Table 2

Click here to access/download
Supporting Information
TableS2.xlsx

Supporting Information Figure 1

Click here to access/download
Supporting Information
FigureS1.eps

Supporting Information Figure 2

Click here to access/download
Supporting Information
downcore_slices.png

Supporting Information Figure 3

Click here to access/download
Supporting Information
FigureS3.eps

Supporting Information Figure 4

Click here to access/download
Supporting Information
FigureS4.eps

Supporting Information Figure 5

Click here to access/download

Supporting Information

FigureS5.eps

Supporting Information Figure 6

Click here to access/download
Supporting Information
FigureS6.eps

Supporting Information Figure 7

Click here to access/download
Supporting Information
FigureS7.eps

Supporting Information Figure 8

Click here to access/download
Supporting Information
FigureS8.eps

Reviewer #1: This manuscript addresses how to sample the microbial communities in sediments and has many interesting implications.
However, the major conclusion seems to be that the diversity is not fully sampled, even with three replicates, but that the replicates appear to more similar than samples from different depths. This is a common observation in similar studies with soil and marine sediments. From this perspective, this observation does not appear to be particularly novel. Importantly, the possibility of sequencing errors increasing the observed ASV was not addressed. This is critical since the source of variation in ASVs is central to the major conclusions.

The authors seem to believe that their amplicands are derived from free DNA and not intact bacterial cells in their sediments. Evidence should be provided to support this assumption (Lines 58-64 and elsewhere).

Authors: We agree that eDNA is a complex mixture of living cells, unactive cells, fragments of dead cells and extracellular DNA from a variety of sources including faeces, urine and saliva. We have now added further information to this section. However as shown in previous studies (e.g. Corinaldesi et al) extracellular DNA is likely to be a substantial proportion.

Line 74. Define 'eDNA' at first mention.

Authors: We agree and we have corrected.

Line 75-76. I find it difficult to believe the claim that reproducibility with core slices has not be examined previously. While it may not have been the focus of many papers, it is routine for many laboratories to examine replicates of samples.

Authors: We agree that triplicates are the norm in many environmental studies, however this is not the case in paleolimnology, perhaps due to the large number of samples generated when replicate are used. While some eDNA based paleolimnological studies use duplicates there are a larger number of studies that are conducted on single replicates per depth e.g. Monchamp et al 2016 (Appl Environ Microbiol; vol 82(21): 6472–6482.); Voldstad et al 2020 (Quaternay Science Reviews; vol 234, 106207); Ficetola et al 2018 (Science Advances; vol4, eaar4292); Parduccu et al 2015 (Philosophical Transactions of the Royal Society B; vol 370: 20130382).*. Among the studies that do have duplicates they tend to only use this as a quality check and they have not investigated heterogeneity in the replicates.*

Legend to Figure 1. Please provide the depth and/or age of the four 'clusters' sampled at each site.

Authors: Including the depth or age would mean the inclusion of a very complex legend. We agree that this information should be available and it is given in Supplementary Information 1, we have now referred to this in the figure caption.

Line 225-229. The problem with ASVs based on one bp substitutions is that sequencing errors are included as novel ribotypes. Were any controls performed to estimate the prevalence of sequencing errors?

Authors: We agree that sequencing errors will inflate richness estimations. The DADA2 pipeline includes an error correction of the reads to mitigate the prevalence of errors in the data set. Furthermore, singletons which are likely to be sequencing errors were removed by DADA2. No specific controls were undertaken to detect sequencing errors per se. This is standard practise and this bioinformatic pipeline is now used by most environmental metabarcoding studies worldwide.

Lines 231-246. The assumption that free DNA is being sampled is surprising. In many studies, it is assumed that the DNA comes from intact cells. Have any controls been performed to demonstrate that free DNA is the source of any or most of the amplicands?

Authors: The study by Corinaldesi et al showed that a large proportion (>95%) of DNA in marine sediments is extracellular. Although it may be lower in lake sediments it is still likely to be a substantial proportion of the DNA in the sediment (Ellegaard et al 2020). This is likely to be especially true deeper down in the core where dead cells would have been degraded and many of the bacterial taxa do not form cysts (or similar structures) that limit degradation. It was not our intension to infer that all DNA was extracellular. We have now added a definition of eDNA to clarify this point.

Lines 258-259. Please explain your reasoning further. Ordinarily, I would have expected more bioturbation would lead to more mixing and more shared ASVs between samples from different depths. You seem to have the opposite conclusion.

Authors: We are making the argument that the bioturbation does not affect the replicates equally and that vertical bioturbation may bring ASVs present from other depths into a subset of the replicates and thus affect the number of shared ASVs amongst replicates. However, it could well increase the proportion of shared ASVs amongst depths. We have clarified that we were discussing within slice shared ASVs. It now reads "While lakes Nganoke and Pounui have higher levels of bioturbation, likely reducing the number of shared reads amongst triplicates, there is lower sedimentary layering in Lake Nganoke."

Lines 297-298. Rather than compare by cluster number, the similarity in Figure 4 should be compared either by sample depth or age. Not only would this facilitate comparisons between the lakes, but it would eliminate artifacts due to differences in sampling

Authors: We have now undertaken the analysis by depth. The results were very similar (with the exception that the regression for Paringa which is now significantly positive).

Lines 303-304. The major conclusion of this study seems to be that the diversity is not

fully sampled, even with three replicates, but that the replicates appear to more similar than other samples from different depths. This is a common observation in similar studies with soil and marine sediments. From this perspective, how important is this observation?

Authors: We believe that this is a very important aspect to investigate as very little work has been undertaken on this on downcore sediments. Given the age of these samples we do not believe they are comparable to studies undertaken on surface sediments. Just because things hold true in surface sediments does not mean that they necessarily hold at other sediment depths

In Supplementary table 1, the columns labeled 'age' are incorrect since the year of deposition seems to be the number given. Please either provide the actual age or change the column titles.

Authors: We have amended this.

The manuscript contains many careless grammatical errors and should be carefully proofread. For instance, "Samples were collected from a variety of depths (1-cm width) over a period spanning approximately 1,200 years." [lines 24-25] states that samples were collected over 1200 years not that the samples were 1200 years in age."

Authors: We have now undertaken a careful proofread of the manuscript and corrected a number of grammatical errors. We thank the review for highlighting these.

Reviewer #2: I have now reviewed the manuscript of Pearman et al. entitled "Single samples from lake sediment core slices are sufficient to characterize dominant bacteria communities". From a technical point of view, this study does not present any fatal technical flaw, although some details about methods needs to be clarified (see details below). However, I am perplexed by the purpose and objectives of this manuscript. Many publications on sedimentary ancient DNA insist on the importance of the replication at every step of the workflow (sampling, extraction, PCRs etc.). Variability can be both biological and technical, therefore replication, rather than being a result per se, is often viewed as a quality control necessary to make robust inferences. It allows to assess replicability of results, to identify sources of stochasticity and to potentially identify false positive. In light of this, analyses presented (i.e., rarefaction, homogeneity of the replicates) look like a suite of quality checks, and I am puzzled by the added value of this manuscript in absence of further results.

Importantly, I think the title, presented as the main result in the conclusion, is highly problematic. I am not saying here that authors' conclusions are erroneous (I do believe that one sample is enough to characterize dominant taxa) but it implicitly implies that future studies can use a single sample, which is highly problematic for making robust inference and goes again all the recommendations and guidelines edited in the field during the last 5 years. Replication is a key step of eDNA studies (e.g., Zinger et al. 2019 Molecular Ecology), especially when dealing with ancient DNA where location variation

exists and stochasticity in species detectability is important.

Below is a list of other major issues:
-(i) the first hypothesis is not really one, this is an evidence: the higher the sampling effort, the higher the probability to find rare taxa. This has been described decades ago and is valid for all biodiversity surveys.

Authors: We have changed the hypothesis. They now read: Replication would provide a better representation of the ASV richness within core slices and the richness would be better represent with increasing depth; (2) community structure and composition will be more similar within slices than between slices; and (3) that there would be greater variability in the triplicates with increasing age of sediment due to differences in DNA degradation over time.

-(ii) Hypothesis 2 "diversity and richness would not differ significantly between triplicates within each slice" is not properly assessed. I have not seen any comparison of richness among triplicates. Besides what do authors means here by "diversity": alpha (diversity within sub-sample) or beta (variation in composition between samples)? Results suggest the second but, in this case, the term "community composition" is better adapted.

Authors: We have changed the hypothesis and used the term community structure and composition instead of diversity.

-(iii) Sediment cores from Lake Pounui have been frozen, while others were kept at 4°C. Freezing and thawing is known to damage DNA, which is critical in ancient DNA analyses. Several publications suggest to keep unfrozen sediment dedicated to DNA analyses at 4°C. I am wondering to which extent this may have contributed to the pattern observed in Lake Pounui, for which the similarity between sub-samples from a given layer was the lowest.

Authors: We agree this was a slightly ambiguous statement. It was meant to mean that the core was sampled and the sediment slices were frozen within 12 hours. Not that the core was frozen. We believe that this slight differences in timing of processing would not have affected the results.

-(iv) in absence of data about sample composition etc., it is complicated to properly assess and understand observed patterns. Results about community composition, even in supplementary information, would be interesting.

Authors: We agree and we have added in some more results to the manuscript. However, the main aim of this paper was not to investigate the shifts, and potential reasons for these shift swhich will be presented elsewhere in more depth for each lake. The key aspect of this study was to look at the variability in downcore replication across several lakes with a variety of conditions.

-(v) more information about ASV's retrieved in controls would be welcomed to show that the information inferred really from ancient DNA, and not modern contamination.

Authors: We have now added a supplementary table with the number of reads in the controls and the effect this had on the reads in the samples when removed.

Minor comments:
-I think there is a typo in the "Ethics statement" section: "Department of Conversation" is likely "Department of Conservation".

Authors: Corrected

Abstract:
-line 21: "but further validation is required", this sentence is vague. Can you please precise which aspects need to be further validated.

Authors: We agree and this now reads "...but further studies investigating factors such as sample heterogeneity and DNA degradation are required.."

-line 25-26: this sentence is a bit ambiguous, it is unclear if triplicates were undertaken at each step or just for sampling. Please reformulate.

Authors: Corrected

Introduction:
-line 49: more precisely, metabarcoding is "the identification of taxa from samples containing DNA from a mix of species".

Authors: We have now amended as suggested

-line 58-64: you could also cite DNA degradation processes here.
We agree and have now amended as suggested

-line 71: "little replication has been undertaken when extracting DNA from lake sediment". I am not sure what do authors mean here? Do you mean that little studies have investigated the effect of sampling replicates? Many studies cited by the authors acknowledge the importance of replication, including at the sampling step (e.g., Domaizon et al. 2017).

Authors: The studies by Capo and Domaizon use duplicates. But many other studies just use a single sample from a slice. E.g. Monchamp et al 2016 (Appl Environ Microbiol. 2016 Nov 1; 82(21): 6472–6482.); Voldstad et al 2020 (Quaternay Science Reviews vol 234); Ficetola et al 2018 (science advances vol4, eaar4292); Parducu et al 2015 (Philosophical transcations of the Royal Society B). We thus believe that an investigation using triplicates with depth and looking at the variability and trends was required and add new information to the rapidly expanding area of research in paleolimnology.

-lines76-78: this sentence is not really correct; many sedimentary ancient DNA studies use extraction replicates, which generally implies sub-sampling at each depth. In some cases, well-mixed composite samples could also be used to attenuate the sub-sampling effect (or even better, multiple extractions from composite samples).

Authors: As stated in the comment above there has been little research on variability in sediment core replicability. While well mixed composite samples could mitigate any issues it does not solve the issue of a lack of knowledge about the variability within sediment slices

-lines 82-83: see comment above about hypothesis 1

Authors: We have changed the hypothesis as suggested.

-lines 83-84: the second hypothesis is not properly tested here (see comment above).

Authors: We have changed the hypothesis as suggested.

Material and methods:
-line 142-144: Were these rooms physically separated? How did you clean/sterilized the tools?

Authors: Yes they were, we have now clarified this in the methods section.

-line 154-156: What is the length range of this DNA fragment? For this type of analyses, small fragments are generally preferred. Indeed, DNA degradation reduces the probability to find long fragments.

Authors: The amplicon is ~400 bp. We agree that that DNA degradation reduces the chances of detecting longer amplicons. However, compared to various studies we are going back a relatively short time (~1000 years) and can readily amplify bacteria throughout the core although as discussed DNA degradation may reduce the diversity of the community.

-line 163: Each step of a sedimentary ancient DNA workflow induces its part of stochasticity. PCR amplification is one of them. Authors amplified each sub-sampling in triplicate but it would have been interesting to sequence them individually to compare the part of variation due to sub-sampling and those due to the PCR amplification process.

Authors: We thank the reviewer for this suggestion and while we agree this is a good idea, it would have added considerable cost and would not have addressed the hypothesis of this study. We will consider this for future studies.

-line 180-181: What was the rationale for using different maxEE for forward and reverse primers?

Authors: A slightly higher maxEE was used for the reverse primer as the reverse sequences had a slightly lower sequence quality compared to the forward as is typical of Illumina Miseq sequencing.

-line 184: "remaining paired-end reads were merged" instead of " remaining reads were merged "

Authors: Changed as suggested.

-line 188-192: Consider adding a supplementary figure showing community composition.
Authors: Now included as suggested.

-Line 192: Please indicate the contamination level of samples (number of contaminating OTUs etc.), this is important for ancient DNA studies.

Authors: We have now included this information.

-line 194-207: Please describe in more details what is the purpose of each of the statistical analyses.

Authors: We have added in some details as to what each analysis was aimed to investigate

-line 200: The notion of "cluster" here and throughout the manuscript is misleading. "Clusters" are generally defined analytically based on similarity between samples, here it seems authors use this term arbitrarily to group replicates from a given sediment layer. For instance, in figure 3, the fact that multiple clusters share the same name can suggest that a similar community composition between lakes. I suggest using another term (e.g. "replicates of a same layer").

Authors: We used this term because the sampling was not undertaken at the same depth/age across the three lakes. We have now changed it to slices in the manuscript and figure 4 and other supplementary plots are now plotted against depth

-line 205-207: This sentence is a bit unclear, please reformulate.
Authors: Changed as suggested.

Results and discussion:
-line 219-220: Both large sampling and sequencing efforts are necessary.

Authors: Changed as suggested

-line 224-226: Authors should also acknowledge that it is often complicated to decipher between genuine rare sequences and errors resulting from PCR and/or sequencing process.

Authors: We agree and have now added this to the paragraph.

-line 241-243: Can you please provide evidence of this (e.g., examples or reference).

Authors: We have added in the community composition were to indicate that the community changed.

-line 243-246: many publications suggest actually the increase replicability at each step, and avoiding pooling to assess replicability of results, define sources of stochasticity and potentially identify false positive.

Authors: we agree and have deleted pooling.

-line 252-254: See my comment above about the preservation of sediment cores from Lake Pounui and potential downstream effects.

Authors: Pounui was not frozen until after samples were collected. We have amended this sentence.

-line 254-256: True but it should also mix the signal between layers.

Authors: We agree and this is note in the text "With lower levels of bioturbation, the stratigraphy would be less disturbed leading to more homogeneous replicates at any depth and consequently a higher shared number of reads."

-line 256-259: This bioturbation by mussels is more likely to induce horizontal mixing than vertical one. I agree that vertical mixing will disturb the stratigraphy, which in turn induce a higher heterogeneity between replicates of a same layer, but one could argue that horizontal mixing would actually lead to a higher homogeneity between replicates.

Authors: The size of a mussel is larger than the slice of sediment used in this study so any bioturbation will induce vertical mixing.

-line 260-262: unclear, please reformulate.

Authors: Changed

-line 270: For the sake of transparency, could you please provide this "not shown" data in Supplementary Information.

Authors: Figure now added included

-line 281: Such variation can be due to biological variation (i.e. sediment heterogeneity) but can also be of technical origin (i.e., stochasticity at each step of the process). This should be mentioned.

Authors: We agree and we have now amended the text

-Line 288-291: Contrarily to above, community can also be simply biologically similar.

Authors: We thank the reviewer for this suggestion. But given that the other two replicates are quite distinct we believe this is unlikely to be this explanation.

-Line 294-299: These results do not support your hypotheses but should be discussed. What can explain the observed pattern.

Authors: We have added in a section about sampling across laminae which is likely to account for the increased variability in the Lake Pounui sediment core.

-Figure 3: It would be interesting to relate patterns observed in Figure 3 with sampling depth, not in term of replicability (Fig. 4) but in term of community change from one layer to the other.

Authors: We agree and have changed as suggested.
