# Supplementary material for: Investigating variability in microbial community composition in replicate environmental DNA samples down lake sediment cores
Source: PLoS One. 2021 May 3;16(5):e0250783. doi: 10.1371/journal.pone.0250783 (PMC8092796; doi:10.1371/journal.pone.0250783)
Supplement: Attachment — Submitted filename: Reviewercomments-v2.docx [file pone.0250783.s011.docx]

Reviewer #1: This manuscript addresses how to sample the microbial communities in sediments and has many interesting implications.
However, the major conclusion seems to be that the diversity is not fully sampled, even with three replicates, but that the replicates appear to more similar than samples from different depths. This is a common observation in similar studies with soil and marine sediments. From this perspective, this observation does not appear to be particularly novel. Importantly, the possibility of sequencing errors increasing the observed ASV was not addressed. This is critical since the source of variation in ASVs is central to the major conclusions.

The authors seem to believe that their amplicands are derived from free DNA and not intact bacterial cells in their sediments. Evidence should be provided to support this assumption (Lines 58-64 and elsewhere).

*Authors: We agree that eDNA is a complex mixture of living cells, unactive cells, fragments of dead cells and extracellular DNA from a variety of sources including faeces, urine and saliva. We have now added further information to this section. However as shown in previous studies (e.g. Corinaldesi et al) extracellular DNA is likely to be a substantial proportion.*

Line 74. Define ‘eDNA’ at first mention.

*Authors: We agree and we have corrected.*

Line 75-76. I find it difficult to believe the claim that reproducibility with core slices has not be examined previously. While it may not have been the focus of many papers, it is routine for many laboratories to examine replicates of samples.

*Authors: We agree that triplicates are the norm in many environmental studies, however this is not the case in paleolimnology, perhaps due to the large number of samples generated when replicate are used. While some eDNA based paleolimnological studies use duplicates there are a larger number of studies that are conducted on single replicates per depth* e.g. Monchamp et al 2016 (Appl Environ Microbiol; vol 82(21): 6472–6482.); Voldstad et al 2020 (Quaternay Science Reviews; vol 234, 106207); Ficetola et al 2018 (Science Advances; vol4, eaar4292); Parduccu et al 2015 (Philosophical Transactions of the Royal Society B; vol 370: 20130382).*. Among the studies that do have duplicates they tend to only use this as a quality check and they have not investigated heterogeneity in the replicates.*

Legend to Figure 1. Please provide the depth and/or age of the four ‘clusters’ sampled at each site.

*Authors: Including the depth or age would mean the inclusion of a very complex legend. We agree that this information should be available and it is given in Supplementary Information 1, we have now referred to this in the figure caption.*

Line 225-229. The problem with ASVs based on one bp substitutions is that sequencing errors are included as novel ribotypes. Were any controls performed to estimate the prevalence of sequencing errors?

*Authors: We agree that sequencing errors will inflate richness estimations. The DADA2 pipeline includes an error correction of the reads to mitigate the prevalence of errors in the data set. Furthermore, singletons which are likely to be sequencing errors were removed by DADA2. No specific controls were undertaken to detect sequencing errors per se. This is standard practise and this bioinformatic pipeline is now used by most environmental metabarcoding studies worldwide.*

Lines 231-246. The assumption that free DNA is being sampled is surprising. In many studies, it is assumed that the DNA comes from intact cells. Have any controls been performed to demonstrate that free DNA is the source of any or most of the amplicands?

*Authors: The study by Corinaldesi et al showed that a large proportion (>95%) of DNA in marine sediments is extracellular. Although it may be lower in lake sediments it is still likely to be a substantial proportion of the DNA in the sediment (Ellegaard et al 2020). This is likely to be especially true deeper down in the core where dead cells would have been degraded and many of the bacterial taxa do not form cysts (or similar structures) that limit degradation. It was not our intension to infer that all DNA was extracellular. We have now added a definition of eDNA to clarify this point.*

Lines 258-259. Please explain your reasoning further. Ordinarily, I would have expected more bioturbation would lead to more mixing and more shared ASVs between samples from different depths. You seem to have the opposite conclusion.

*Authors: We are making the argument that the bioturbation does not affect the replicates equally and that vertical bioturbation may bring ASVs present from other depths into a subset of the replicates and thus affect the number of shared ASVs amongst replicates. However, it could well increase the proportion of shared ASVs amongst depths. We have clarified that we were discussing within slice shared ASVs. It now reads “While lakes Nganoke and Pounui have higher levels of bioturbation, likely reducing the number of shared reads amongst triplicates, there is lower sedimentary layering in Lake Nganoke.”*

Lines 297-298. Rather than compare by cluster number, the similarity in Figure 4 should be compared either by sample depth or age. Not only would this facilitate comparisons between the lakes, but it would eliminate artifacts due to differences in sampling

*Authors: We have now undertaken the analysis by depth. The results were very similar (with the exception that the regression for Paringa which is now significantly positive).*

Lines 303-304. The major conclusion of this study seems to be that the diversity is not fully sampled, even with three replicates, but that the replicates appear to more similar than other samples from different depths. This is a common observation in similar studies with soil and marine sediments. From this perspective, how important is this observation?

*Authors: We believe that this is a very important aspect to investigate as very little work has been undertaken on this on downcore sediments. Given the age of these samples we do not believe they are comparable to studies undertaken on surface sediments. Just because things hold true in surface sediments does not mean that they necessarily hold at other sediment depths*

In Supplementary table 1, the columns labeled ‘age’ are incorrect since the year of deposition seems to be the number given. Please either provide the actual age or change the column titles.

*Authors: We have amended this.*

The manuscript contains many careless grammatical errors and should be carefully proofread. For instance, “Samples were collected from a variety of depths (1-cm width) over a period spanning approximately 1,200 years.” [lines 24-25] states that samples were collected over 1200 years not that the samples were 1200 years in age.“

*Authors: We have now undertaken a careful proofread of the manuscript and corrected a number of grammatical errors. We thank the review for highlighting these.*

Reviewer #2: I have now reviewed the manuscript of Pearman et al. entitled "Single samples from lake sediment core slices are sufficient to characterize dominant bacteria communities". From a technical point of view, this study does not present any fatal technical flaw, although some details about methods needs to be clarified (see details below). However, I am perplexed by the purpose and objectives of this manuscript.
Many publications on sedimentary ancient DNA insist on the importance of the replication at every step of the workflow (sampling, extraction, PCRs etc.). Variability can be both biological and technical, therefore replication, rather than being a result per se, is often viewed as a quality control necessary to make robust inferences. It allows to assess replicability of results, to identify sources of stochasticity and to potentially identify false positive. In light of this, analyses presented (i.e., rarefaction, homogeneity of the replicates) look like a suite of quality checks, and I am puzzled by the added value of this manuscript in absence of further results.

Importantly, I think the title, presented as the main result in the conclusion, is highly problematic. I am not saying here that authors' conclusions are erroneous (I do believe that one sample is enough to characterize dominant taxa) but it implicitly implies that future studies can use a single sample, which is highly problematic for making robust inference and goes again all the recommendations and guidelines edited in the field during the last 5 years. Replication is a key step of eDNA studies (e.g., Zinger et al. 2019 Molecular Ecology), especially when dealing with ancient DNA where location variation exists and stochasticity in species detectability is important.

Below is a list of other major issues:
-(i) the first hypothesis is not really one, this is an evidence: the higher the sampling effort, the higher the probability to find rare taxa. This has been described decades ago and is valid for all biodiversity surveys.

*Authors: We have changed the hypothesis. They now read: Replication would provide a better representation of the ASV richness within core slices and the richness would be better represent with increasing depth; (2) community structure and composition will be more similar within slices than between slices; and (3) that there would be greater variability in the triplicates with increasing age of sediment due to differences in DNA degradation over time.*

-(ii) Hypothesis 2 "diversity and richness would not differ significantly between triplicates within each slice" is not properly assessed. I have not seen any comparison of richness among triplicates. Besides what do authors means here by "diversity": alpha (diversity within sub-sample) or beta (variation in composition between samples)? Results suggest the second but, in this case, the term "community composition" is better adapted.

*Authors: We have changed the hypothesis and used the term community structure and composition instead of diversity.*

-(iii) Sediment cores from Lake Pounui have been frozen, while others were kept at 4°C. Freezing and thawing is known to damage DNA, which is critical in ancient DNA analyses. Several publications suggest to keep unfrozen sediment dedicated to DNA analyses at 4°C. I am wondering to which extent this may have contributed to the pattern observed in Lake Pounui, for which the similarity between sub-samples from a given layer was the lowest.

*Authors: We agree this was a slightly ambiguous statement. It was meant to mean that the core was sampled and the sediment slices were frozen within 12 hours. Not that the core was frozen. We believe that this slight differences in timing of processing would not have affected the results.*

-(iv) in absence of data about sample composition etc., it is complicated to properly assess and understand observed patterns. Results about community composition, even in supplementary information, would be interesting.

*Authors: We agree and we have added in some more results to the manuscript. However, the main aim of this paper was not to investigate the shifts, and potential reasons for these shift swhich will be presented elsewhere in more depth for each lake. The key aspect of this study was to look at the variability in downcore replication across several lakes with a variety of conditions.*

-(v) more information about ASV's retrieved in controls would be welcomed to show that the information inferred really from ancient DNA, and not modern contamination.

*Authors: We have now added a supplementary table with the number of reads in the controls and the effect this had on the reads in the samples when removed.*


Minor comments:
-I think there is a typo in the "Ethics statement" section: "Department of Conversation" is likely "Department of Conservation".

*Authors: Corrected*

Abstract:
-line 21: "but further validation is required", this sentence is vague. Can you please precise which aspects need to be further validated.

*Authors: We agree and this now reads “...*but further studies investigating factors such as sample heterogeneity and DNA degradation are required..”

-line 25-26: this sentence is a bit ambiguous, it is unclear if triplicates were undertaken at each step or just for sampling. Please reformulate.

*Authors: Corrected*

Introduction:
-line 49: more precisely, metabarcoding is "the identification of taxa from samples containing DNA from a mix of species".

*Authors: We have now amended as suggested*

-line 58-64: you could also cite DNA degradation processes here.
*We agree and have now amended as suggested*

-line 71: "little replication has been undertaken when extracting DNA from lake sediment". I am not sure what do authors mean here? Do you mean that little studies have investigated the effect of sampling replicates? Many studies cited by the authors acknowledge the importance of replication, including at the sampling step (e.g., Domaizon et al. 2017).

*Authors: The studies by Capo and Domaizon use duplicates. But many other studies just use a single sample from a slice. E.g. Monchamp et al 2016 ([Appl Environ Microbiol](https://www.ncbi.nlm.nih.gov/pmc/articles/PMC5066364/). 2016 Nov 1; 82(21): 6472–6482.); Voldstad et al 2020 (Quaternay Science Reviews vol 234); Ficetola et al 2018 (science advances vol4, eaar4292); Parduccu et al 2015 (Philosophical transcations of the Royal Society B). We thus believe that an investigation using triplicates with depth and looking at the variability and trends was required and add new information to the rapidly expanding area of research in paleolimnology.*

-lines76-78: this sentence is not really correct; many sedimentary ancient DNA studies use extraction replicates, which generally implies sub-sampling at each depth. In some cases, well-mixed composite samples could also be used to attenuate the sub-sampling effect (or even better, multiple extractions from composite samples).

*Authors: As stated in the comment above there has been little research on variability in sediment core replicability. While well mixed composite samples could mitigate any issues it does not solve the issue of a lack of knowledge about the variability within sediment slices*

-lines 82-83: see comment above about hypothesis 1

*Authors: We have changed the hypothesis as suggested.*

-lines 83-84: the second hypothesis is not properly tested here (see comment above).

*Authors: We have changed the hypothesis as suggested.*

Material and methods:
-line 142-144: Were these rooms physically separated? How did you clean/sterilized the tools?

*Authors: Yes they were, we have now clarified this in the methods section.*

-line 154-156: What is the length range of this DNA fragment? For this type of analyses, small fragments are generally preferred. Indeed, DNA degradation reduces the probability to find long fragments.

*Authors: The amplicon is* ~400 bp. We agree that that DNA degradation reduces the chances of detecting longer amplicons. However, compared to various studies we are going back a relatively short time (~1000 years) and can readily amplify bacteria throughout the core although as discussed DNA degradation may reduce the diversity of the community.

-line 163: Each step of a sedimentary ancient DNA workflow induces its part of stochasticity. PCR amplification is one of them. Authors amplified each sub-sampling in triplicate but it would have been interesting to sequence them individually to compare the part of variation due to sub-sampling and those due to the PCR amplification process.

*Authors: We thank the reviewer for this suggestion and while we agree this is a good idea, it would have added considerable cost and would not have addressed the hypothesis of this study. We will consider this for future studies.*

-line 180-181: What was the rationale for using different maxEE for forward and reverse primers?

*Authors: A slightly higher maxEE was used for the reverse primer as the reverse sequences had a slightly lower sequence quality compared to the forward as is typical of Illumina Miseq sequencing.*

-line 184: "remaining paired-end reads were merged" instead of " remaining reads were merged "

*Authors: Changed as suggested.*

-line 188-192: Consider adding a supplementary figure showing community composition.
*Authors: Now included as suggested.*

-Line 192: Please indicate the contamination level of samples (number of contaminating OTUs etc.), this is important for ancient DNA studies.

*Authors: We have now included this information.*

-line 194-207: Please describe in more details what is the purpose of each of the statistical analyses.

*Authors: We have added in some details as to what each analysis was aimed to investigate*

-line 200: The notion of "cluster" here and throughout the manuscript is misleading. "Clusters" are generally defined analytically based on similarity between samples, here it seems authors use this term arbitrarily to group replicates from a given sediment layer. For instance, in figure 3, the fact that multiple clusters share the same name can suggest that a similar community composition between lakes. I suggest using another term (e.g. "replicates of a same layer").

*Authors: We used this term because the sampling was not undertaken at the same depth/age across the three lakes. We have now changed it to slices in the manuscript and figure 4 and other supplementary plots are now plotted against depth*

-line 205-207: This sentence is a bit unclear, please reformulate.
*Authors: Changed as suggested.*

Results and discussion:
-line 219-220: Both large sampling and sequencing efforts are necessary.

*Authors: Changed as suggested*

-line 224-226: Authors should also acknowledge that it is often complicated to decipher between genuine rare sequences and errors resulting from PCR and/or sequencing process.

*Authors: We agree and have now added this to the paragraph.*

-line 241-243: Can you please provide evidence of this (e.g., examples or reference).

*Authors: We have added in the community composition were to indicate that the community changed.*

-line 243-246: many publications suggest actually the increase replicability at each step, and avoiding pooling to assess replicability of results, define sources of stochasticity and potentially identify false positive.

*Authors: we agree and have deleted pooling.*

-line 252-254: See my comment above about the preservation of sediment cores from Lake Pounui and potential downstream effects.

*Authors: Pounui was not frozen until after samples were collected. We have amended this sentence.*

-line 254-256: True but it should also mix the signal between layers.

*Authors: We agree and this is note in the text “With lower levels of bioturbation, the stratigraphy would be less disturbed leading to more homogeneous replicates at any depth and consequently a higher shared number of reads.”*

-line 256-259: This bioturbation by mussels is more likely to induce horizontal mixing than vertical one. I agree that vertical mixing will disturb the stratigraphy, which in turn induce a higher heterogeneity between replicates of a same layer, but one could argue that horizontal mixing would actually lead to a higher homogeneity between replicates.

*Authors: The size of a mussel is larger than the slice of sediment used in this study so any bioturbation will induce vertical mixing.*

-line 260-262: unclear, please reformulate.

*Authors: Changed*

-line 270: For the sake of transparency, could you please provide this "not shown" data in Supplementary Information.

*Authors: Figure now added included*

-line 281: Such variation can be due to biological variation (i.e. sediment heterogeneity) but can also be of technical origin (i.e., stochasticity at each step of the process). This should be mentioned.

*Authors: We agree and we have now amended the text*

-Line 288-291: Contrarily to above, community can also be simply biologically similar.

*Authors:* We thank the reviewer for this suggestion. But given that the other two replicates are quite distinct we believe this is unlikely to be this explanation.

-Line 294-299: These results do not support your hypotheses but should be discussed. What can explain the observed pattern.

*Authors: We have added in a section about sampling across laminae which is likely to account for the increased variability in the Lake Pounui sediment core.*

-Figure 3: It would be interesting to relate patterns observed in Figure 3 with sampling depth, not in term of replicability (Fig. 4) but in term of community change from one layer to the other.

*Authors: We agree and have changed as suggested.*
